# A Summary of One Research Team’s Contributions to Understanding Physical Activity Behavior in Children and Youth

**DOI:** 10.3390/ijerph192114136

**Published:** 2022-10-29

**Authors:** Russell R. Pate, Rod K. Dishman, Marsha Dowda, Kerry L. McIver, Karin A. Pfeiffer, Dwayne E. Porter, Ruth P. Saunders, Dianne S. Ward

**Affiliations:** 1 Public Health Research Center, Department of Exercise Science, University of South Carolina, 921 Assembly Street, Suite 212, Columbia, SC 29208, USA; 2 Department of Kinesiology, The University of Georgia Ramsey Student Center, 330 River Road, Athens, GA 30602, USA; 3 Department of Kinesiology, Michigan State University, 308 West Circle Drive, 27R Intramural Rec Sports-Circle, East Lansing, MI 48824, USA; 4 Department of Environmental Health Sciences, Arnold School of Public Health, University of South Carolina, 915 Green Street, Suite 518, Columbia, SC 29208, USA; 5 Public Health Research Center, Department of Health Promotion, Education, and Behavior, University of South Carolina, 921 Assembly Street, Suite 212, Columbia, SC 29208, USA; 6 Department of Nutrition, Gillings School of Global Public Health, University of North Carolina at Chapel Hill, 135 Dauer Drive, CB # 7461, Chapel Hill, NC 27599, USA

**Keywords:** physical activity, young children, children, adolescents, physical activity measurement, physical activity determinants, school-based physical activity interventions, race/ethnicity and physical activity

## Abstract

Schools are well-positioned to provide physical activity opportunities to help youth achieve the recommended 60 or more daily minutes of moderate-to-vigorous physical activity. The Children’s Physical Activity Research Group (CPARG) at the University of South Carolina has focused on understanding physical activity in school-aged youth for 30+ years. The purpose of this article was to critically review (CPARG) contributions to the field in school settings and school-age youth. We reviewed 127 published CPARG articles from six research projects conducted between 1993–2019. The review was guided by questions in five categories: measurement of physical activity and its determinants, characteristics of physical activity behavior, correlates/determinants of physical activity, physical activity interventions, and race/ethnicity and physical activity. Results were summarized by question and synthesized across categories. CPARG contributions included assessing physical activity levels, patterns, forms, and contexts; identifying and measuring physical activity correlates/determinants; and conducting school-based physical activity interventions. Identifying multiple domains of physical activity determinants enables researchers and practitioners to select/design age-appropriate, valid, and reliable instruments to assess determinants. Focusing on determinants enables them to create effective physical activity interventions, environments, programs, and policies in schools. These efforts must address race/ethnicity differences, ensuring that measurement instruments and intervention strategies are culturally appropriate.

## 1. Introduction

Higher levels of physical activity are related to better health, social, emotional, cognitive, and academic outcomes in children and adolescents [1]. To achieve these benefits, the United States Department of Health and Human Services has issued physical activity guidelines for school-aged children and adolescents that call for youth to engage daily in 60 or more minutes of moderate-to-vigorous physical activity [2]. Yet, fewer than one-quarter of children and youth ages 6–17 meet this physical activity guideline [3], and compliance with the guideline decreases as children transition into adolescence [4,5,6].

Public health interventions can reduce the age-related decline in physical activity in U.S. youth [1]. Because schools are well-positioned to provide organized and free-time physical activity opportunities and to help children and adolescents accumulate 60 min of moderate-to-vigorous physical activity each day [7], they have played an important role in promoting youth physical activity. Helping schools promote physical activity is ideally based on an understanding of factors that influence physical activity throughout childhood and adolescence.

Accordingly, the Children’s Physical Activity Research Group (CPARG) at the University of South Carolina has focused much of its research on understanding physical activity in schools and school-age youth. CPARG, which is led by Russell R. Pate and based in the Arnold School of Public Health’s Department of Exercise Science, is an interdisciplinary team of faculty investigators, staff, post-doctoral fellows, and students who share a common interest in helping children become more physically active. The group originated in the early 1990s, with studies funded by the Centers for Disease Control and Prevention and American Heart Association, and has been funded continuously by NIH since 1993 to undertake a range of research projects related to physical activity in youth [8,9].

Most reviews of the scientific literature on a particular topic are based on research published by multiple investigative teams. That traditional strategy is well established and provides important contributions to science. The review presented in this article applies a method that is used less frequently, but which also contributes valuable information to the body of knowledge. This review focuses on a single research team and a subset of its body of work conducted over a period of more than three decades. This team has had stable membership and has been productive in terms of funding and publishing across that entire time. Many of the published articles have been widely cited in the literature. The group’s work has consistently focused on expanding the body of knowledge on physical activity behavior in children and youth, and most of its studies have been conducted in educational settings, including childcare centers, elementary schools, middle schools, high schools, and afterschool programs. The research team has been interdisciplinary throughout its lengthy history, and its studies have benefited from expertise in exercise science, experimental design and biostatistics, measurement, health behavior, physical activity environments, Geographic Information Systems, process evaluation, educational practice, nursing, and public health. In addition to describing CPARG’s work, this review reflects how research on a specific topic, physical activity in children and youth, has evolved over time, both conceptually and methodologically. In addition, the review, while limited by its subjective and qualitative nature, benefits from the research team’s deep familiarity with the studies and their findings.

This paper synthesizes findings from multiple articles that used varying methodologies in different settings with diverse samples, providing a perspective on children’s physical activity behavior that is both broad and deep. The focused synthesis of work completed over decades elucidates patterns not readily evident with a single article and therefore adds value beyond the impact of the individual articles published previously.

The purpose of this article is to provide a summary and critical review of CPARG’s contributions to the body of knowledge on understanding physical activity behavior in children and youth, guided by the goal of summarizing and critically examining findings on:

1. Methods for measurement of physical activity behavior and determinants of physical activity behavior.
2. Characteristics of physical activity behavior in children and youth.
3. Determinants and correlates of physical activity in children and youth.
4. Effectiveness of school-based interventions to promote physical activity in children and youth.
5. Race/ethnicity and physical activity in children and youth.

### Overview of the Studies

CPARG has engaged in intervention and observational studies in childcare centers and elementary, middle, and high school students (see Table 1). Completed CPARG projects that are relevant to school physical activity interventions include Active Winners, Lifestyle Education for Activity Program (LEAP), Trial of Activity in Adolescent Girls (TAAG), Children’s Activity and Movement in Preschool Study (CHAMPS), Study of Health and Activity in Preschool Environments (SHAPES), and Transitions and Activity Changes in Kids (TRACK). Active Winners was a physical activity afterschool and summer intervention in middle school boys and girls with data collection in elementary and middle schools. LEAP was a high school physical activity intervention study with girls; data collection took place in middle and high schools. TAAG was a middle school physical activity intervention study for girls with data collection in middle schools. CHAMPS was an observational study with data collection in childcare centers. SHAPES was physical activity intervention for preschool-aged children with data collection in childcare centers. TRACK was an observational cohort study of physical activity and multiple domains of determinants of physical activity with data collection in elementary, middle, and high schools.

The journal articles featured in this manuscript focus on physical activity in school-aged children with an emphasis on school settings or applications to school settings. CPARG has also examined home and community settings within the projects listed in Table 1 and in additional projects, such as the Healthy Communities Study (2010–2015), FitnessGram Project (2015–1019), and LAUNCH (2017–2022). The Healthy Communities Study was a multicenter study that examined the influence of programs and policies in communities across the United States on children’s eating, physical activity, and weight status [10]. The SC FitnessGram Project involved a collaboration with the SC Department of Health and Environmental Control, the SC Department of Education, and the Blue Cross Blue Shield of SC Foundation [11]. CPARG’s role was to analyze data collected by public school personnel and provided to CPARG by the SC Department of Education. LAUNCH (Linking Activity, Nutrition, and Child Health) is a current and on-going observational study to examine the relationships among physical activity, nutrition, and weight status in children as they develop from infancy to preschool age [12].

## 2. Materials and Methods

We conducted a critical review of completed work relevant to school settings published by members of CPARG from the projects Active Winners, LEAP, TAAG, SHAPES, CHAMPS, and TRACK, conducted between 1993–2019. The review and synthesis of findings took place in six steps:

Step 1. We compiled published articles for each of six projects funded and conducted from 1993 to 2019. Manuscript preparation and submission continued beyond the period of active funding in each project.

Step 2. We assigned articles into categories based on the primary focus of the article: Measurement of Physical Activity and Determinants, Physical Activity Behavior, Correlates/Determinants of Physical Activity, Physical Activity Interventions, Adiposity, and Other (motor skills, child perspectives, risk behaviors, diet, and depression). The first four categories are focal CPARG areas and are included in the critical review reported in this paper. The “Adiposity” (7 papers) and “Other” (13 papers) categories were excluded. We also identified another important category embedded within the four focal CPARG categories: Race/Ethnicity and Physical Activity. Thus, five core CPARG categories are included in this manuscript. We documented the number of citations to date for each article (Google Scholar).

Step 3. We extracted the setting, methods, and key findings from each article into five tables:

- Measurement of Physical Activity and Determinants
- Physical Activity Behavior
- Correlates/Determinants of Physical Activity
- Physical Activity Interventions
- Race/Ethnicity and Physical Activity

Step 4. After a preliminary review of the content, we devised a set of guiding questions specific to each of the five categories for the critical review (see Table 2). The guiding questions for correlates and determinants of physical activity reflect CPARG’s emphasis on developmental/transitional phases in children’s physical activity. In this category, we selected published articles with longitudinal designs, multiple domains of influences, high numbers of citations, high relevance to school settings, and/or those presenting findings of primary study aims.

Step 5. We conducted a critical review of findings for each of the five categories and summarized results based on the guiding questions for that category.

Step 6. We synthesized findings across categories.

## 3. Results

**Figure 1:**
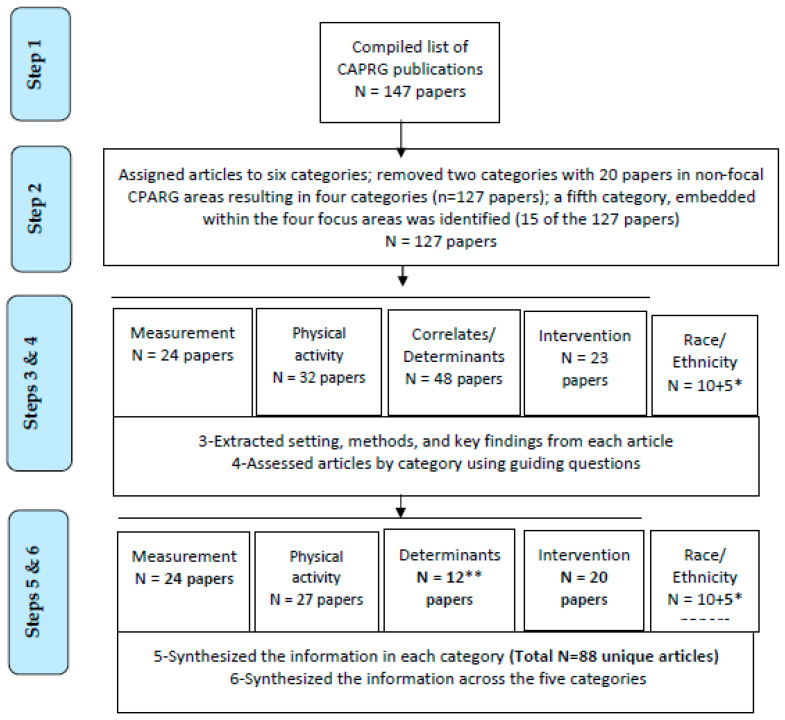
CPARG Publications by Category of Analysis. * 15 papers were drawn from the other four categories (10 duplicate & 5 unique to Race/Ethnicity); ** This subset of papers consists of studies that were longitudinal, included multiple domains of influences, and/or were reports of primary study aims (i.e., focused on determinants versus correlates).

The CPARG articles reflected in each of the six steps of the process are presented in Figure 1. As shown in the figure, the critical review reported in this study was based on 88 unique published CPARG articles.

The results for each of the five reviews are presented below. Table 3 presents how each of the CPARG projects contributed to the five review categories. A complete listing of CPARG publications for each of the six research projects is provided as a Supplementary Materials File S1.

### 3.1. Findings Related to Methods for Measurement of Physical Activity Behavior and Influences on Physical Activity Behavior

Twenty-four articles from five projects (there were no SHAPES measurement papers) pertained to measurement of physical activity or measurement of the determinants of physical activity in children and adolescents. A variety of measurement methods for assessing physical activity are reflected in CPARG’s work, which began in the early 1990s. Accelerometers were not available to be used in large studies until the early to mid-2000s; for example, the National Health and Nutrition Examination Survey (NHANES) began using accelerometers in the 2003–2006 cycle [13]. Young children cannot self-report their physical activity, which necessitated observation or parent report in the pre-accelerometer days. Plus, each method of physical activity measurement offers both advantages and disadvantages. For example, accelerometers are not subject to recall biases, but self-report measures can capture important contextual information. Furthermore, cut points for accelerometers need to be age appropriate and differ from instrument to instrument.

#### 3.1.1. Measurement of Physical Activity in Preschool-Aged Children

CPARG investigators developed three instruments, reported in three articles [14,15,16], to observe physical activity and to record contextual information related to physical activity behavior. Prior instruments, such as CARS [17], had assessed only physical activity. The Observational System for Recording Physical Activity in Children-Preschool Version (OSRAC-P) recorded physical activity in preschool-aged children in the childcare setting, with coding for the behaviors sit and squat, lie down, stand, and walk and the indoor and outdoor social and non-social contexts [14]. The OSRAC-H recorded physical activity and social context in the children’s home [15], and the OSRAC-E developed a system to observe levels and types of physical activity and social contexts in elementary school children during the school day [16]. These papers have been cited more than 300, 90, and 20 times, respectively, for the childcare center, home, and elementary school versions. These methods have also been modified for use with children with developmental delays (OSRAC-DD) [18] and in youth sports (OSRAC-YS) [19].

Six articles addressed measuring physical activity in preschool-aged populations using accelerometers. Two papers addressed accelerometer cut points in children 3 to 5 years of age. One of the papers established moderate-to-vigorous physical activity (420 counts/15 s) and vigorous physical activity(842 counts/15 s) cut points for the Actigraph (Fort Walton Beach, FL) [20]. This paper has been cited more than 650 times. The second paper established cut-points for the Actical and has been cited nearly 300 times [21]. Both studies validated the accelerometers with the rate (V) of oxygen (O₂) the body uses during exercise using a portable metabolic system (Cosmed). Two papers addressed measurement concerns, such as number of days of accelerometer monitoring required to collect reliable physical activity [22] and sedentary behavior data [23]. Two additional articles addressed equating cut points from different studies [24] and meeting physical activity guidelines using different cut points from published studies [25].

In summary, CPARG developed and tested observational tools for assessing context-specific physical activity in childcare and elementary school settings that have been widely used and adapted to additional settings and contributed substantially to the literature on accelerometry methodology in preschool-aged children.

#### 3.1.2. Measurement of Physical Activity in School-Aged Children

Three papers addressed the development and measurement properties of self-reported physical activity instruments in school-aged children. In 1997 Weston et al. published the validation of the Previous Day Physical Activity recall (PDPAR) [26], which has been cited more than 500 times. The instrument, which was validated with accelerometry, divided the day into 30-min blocks during the afterschool hours (3:00–11:30 p.m.). Youth reported intensities of very light, light, medium, or hard, which were coded based on metabolic equivalent for task (a unit that estimates the amount of energy used by the body during physical activity) or MET values and added to obtain a score for total physical activity or moderate-to-vigorous physical activity. An extension of the PDPAR, with more than 300 citations, was developed for children to self-report their physical activity over 3 entire days using 30-min time blocks from 7:00 am to midnight (3DPAR) [27]. Participants selected activities from a list of 55 common activities and rated each activity as light, moderate, hard, or very hard. The instrument was validated in 70 8th and 9th grade girls with accelerometers; the paper has received more than 300 citations.

A third paper examined factorial validity and invariance of the 3DPAR among 2 cohorts of 8th grade girls (*n* = 955 and *n* = 1797) [28]. Analyses indicated that the 3DPAR exhibited cross-validity between the cohorts and that the instrument could be used in both Black and White adolescent girls. These instruments have also been validated and used in other countries, including Greece and Singapore [29,30].

CPARG investigators also conducted two accelerometer-based studies of school-aged children. In the first paper in 1996, which has received 1999 citations, the group calibrated the Computer Science and Applications, Inc. (CSA) accelerometer (Model #7164) for children ages 6–18 years. Children walked and ran on a treadmill while wearing the accelerometer; respiratory gas exchange was measured using indirect calorimetry [31]. A regression equation was developed to estimate METs from counts. This equation produced age-specific cut points for different MET levels, such as 4.0 METS for moderate-to-vigorous physical activity and 7.0 METs for vigorous physical activity. This equation was used by NHANES for boys and girls aged 6–17 years and for the TRACK study [5,32].

In the second paper, accelerometer cut points were determined for 8th grade girls; this article has received more than 750 citations [33]. Girls performed 10 common activities that varied in intensity and wore accelerometers, a heart rate monitor, and a Cosmed K4b2 portable metabolic unit for measurement of VO2. A random coefficient model was used to estimate the relationship between accelerometer counts and VO2. The threshold ranges for sedentary, light, moderate, and vigorous physical activity were 0–50, 51–1499, 1500–2600, and >2600 counts per 30 s, respectively.

One study compared multiple methods of assessing physical activity in school-aged children. Using data obtained from 409 boys and girls, Dowda triangulated data from accelerometers, self-report, and parent report of child’s activity [34]. Structural equation models were used to assess the relationship between physical activity and selected correlates of physical activity. The study concluded that combining measures of physical activity from different sources may improve the identification of correlates of physical activity.

Another study assessed the variability in energy expenditure among 74 adolescent girls (13–14 years of age) using indirect calorimetry for ten activities and a submaximal cycle ergometer test to estimate cardiorespiratory fitness [35]. Energy expenditure for common activities was highly variable, especially among low intensity activities.

In summary, CPARG contributed substantially to the assessment of physical activity in school-aged children with self-report instruments that provided valuable contextual information, as well as to the science of assessing physical activity via accelerometers.

#### 3.1.3. Measurement of Correlates/Determinants of Physical Activity in School-Aged Children

Nine articles focused on the development and assessment of validity, reliability, factorial validity, and/or invariance of psychosocial influences on physical activity in children and adolescents. Psychosocial scales assessed included beliefs, motives, self-management strategies, attitudes, outcome expectancies, perceived behavioral control, self-efficacy, enjoyment, self-motivation, social provisions, social influences, subjective norms, and physical self-description related to physical activity.

Two articles reported instruments developed for both boys and girls. Saunders et al. developed and validated a set of instruments using factor analysis with data from a questionnaire administered to 558 predominately Black 5th grade boys and girls [36]. A social influences scale (8 items) contained a single factor, a 17-item self-efficacy scale contained 3 factors (support seeking, barriers, positive alternatives), and a 16-item beliefs scale contained two factors (social outcomes and physical activity outcomes). Reliability coefficients ranged from about 0.50 to 0.78. This paper has been cited more than 300 times. Dishman et al. tested construct validity of scales (beliefs and motives) in 5th and 6th grade boys and girls [37]. Multi-group longitudinal factorial invariance was confirmed between boys and girls, overweight and normal weight children, and non-Hispanic Black and White children.

CPARG developed instruments specifically for girls because two projects, LEAP and TAAG, addressed interventions in girls. Seven articles were published presenting the measurement characteristics of the sets of scales used in these studies. Dishman et al. tested and confirmed factorial invariance and latent mean structure of questionnaires that measured attitudes, self-efficacy, subjective norms, and perceived behavioral control on participation in physical activity in Black and White 8th grade girls [38]. Furthermore, factorial validity and invariance of physical self-description were evaluated and confirmed among 12th grade adolescent Black and White girls [39]. Another paper examined the measurement properties of enjoyment of physical activity in 8th grade adolescent girls and has been cited more than 600 times [40]. Evidence of factorial validity and convergent evidence for construct validity indicated that the Physical Activity Enjoyment Scale (PACES) was a valid measure of physical activity enjoyment and were similar in Black and White girls.

Dishman et al. tested and found evidence for construct validity in measures of self-management strategies, self-efficacy, barriers, outcome expectancies, and enjoyment of physical activity, as well as for self-reported physical activity in 6th and 8th grade girls [41]. Furthermore, the association between self-efficacy and physical activity was mediated by self-management strategies, which suggests the importance of self-management strategies as an intervention focus. This paper has been cited more than 400 times.

Motl et al. found longitudinal evidence of factorial invariance of questions measuring social-cognitive determinants of physical activity among adolescent girls [42]. These variables included unidimensional measures of attitudes, subjective norms, perceived behavioral control, and self-efficacy among adolescent girls and has been cited over 300 times. Similarly social provisions for physical activity among adolescent Black and White girls was found to exhibit construct validity, as well as longitudinal factor invariance [43], as was a 9-item version of a self-motivation for physical activity scale for Black and White adolescent girls [44].

In summary, CPARG contributed substantially to the literature on development of valid and reliable psychosocial scales to measure the determinants of physical activity in school aged children and adolescents, particularly in White and Black girls. Because all scales were grounded in established theory, these articles also contributed to the theory base for determinants and correlates of physical activity in children and adolescents. The measurement invariance of the scales across time provided the necessary evidence for a valid interpretation of change in the variables in longitudinal studies.

### 3.2. Findings Related to Characteristics of Physical Activity Behavior in Children and Youth

Virtually every study conducted by the CPARG investigative team has focused on physical activity, either as an outcome or exposure. Accordingly, every study included one or more measures of physical activity that were applied to a sample of children selected from a target group. The findings of these studies, collectively, provide a comprehensive profile of children’s physical activity and, in some cases, sedentary behavior. A subset of the studies was designed specifically for the purpose of characterizing children’s physical activity behaviors; some of those studies applied cross-sectional study designs, but many of them were longitudinal with the aim focused on determining the pattern for change in physical activity over time. In this section of the paper, we summarize the findings of those 27 studies, some of which measured overall dose of physical activity based on intensity while others were focused on participation in specific forms of physical activity and/or sedentary behavior. The measures of physical activity were diverse, including both self-report and device-based instruments.

#### 3.2.1. Typical Physical Activity Levels and Compliance with Physical Activity Guidelines

As noted above, CPARG has worked extensively in development of accelerometry as an objective measure of physical activity in children and youth. Those measurement tools were then used, in many CPARG studies, to assess physical activity in youth. The findings showed that physical activity levels, quantified as minutes per day at specified intensity levels, were highly variable across children. It was consistently observed that youth spend most of their time in either sedentary behavior or light intensity physical activity [45,46,47]. Moderate-to-vigorous intensity physical activity, the typical target in physical activity guidelines [2], was usually found to be, on average, considerably lower than the 60 min per day dose usually recommended for school-age youth. For example, in a large and diverse sample of 6th grade girls, average moderate-to-vigorous intensity physical activity was 24 + minutes per day and less than half the girls met the recommended level [46]. Similarly, Dowda [48] and Lau [47,49] reported low levels of moderate-to-vigorous physical activity for boys and girls.

CPARG pioneered the use of accelerometry as a measure of physical activity in children of preschool-age (3–5 years) [25,50]. When applied to two independent samples of children attending childcare centers, accelerometry data showed that children were physically active (sum of light, moderate and vigorous intensity) for approximately 15 min per hour of observation. About 50% of children in both samples met a recommendation that children be active for 30 min per hour of their time in the childcare setting [50].

#### 3.2.2. Age-Related Change in Physical Activity and Tracking

Many of CPARG’s studies have employed longitudinal designs and included measures of physical activity that were administered on several occasions across multi-year follow-up periods. The findings of these studies, when considered collectively, provide a comprehensive characterization of age-related change in physical activity in children and youth. In two studies using self-report measures of physical activity, “tracking”, the tendency of persons to stay at a particular ranking within a group, was demonstrated for physical activity in 5th graders transitioning to 7th grade in Active Winners [51] and 8th grade girls transitioning to 12th grade in LEAP [52]. In studies conducted with a diverse sample of girls who were 6th graders at baseline and followed to 8th grade, it was reported, using a device-based measure, that physical activity decreased at a rate of 4% per year [53]. In a cluster analysis of the same sample, girls in clusters that were involved in sports showed less age-related decline in physical activity than cluster of girls characterized by participation in less physically active pursuits [54].

The TRACK study, in its initial phase, followed boys and girls from 5th grade to 7th grade, and then in its second phase, followed the cohort into high school. In both phases of the study, analyses were undertaken to examine age-related change in physical activity. In the cohort followed from 5th to 7th grade it was observed that physical activity had lower tracking coefficients tracked lower in earlier maturing girls [55] than average and later maturing girls and that physical activity declined the most during the school day [49]. A longitudinal class analysis showed that physical activity did not decline with increasing age in high active girls and moderately active boys [56]. When the cohort was followed into high school, a group-based trajectory analysis revealed that some youth remain at a high level of physical activity while transitioning from elementary school to high school [57]. It was also shown, in the total cohort, that physical activity declined more steeply before age 14 than after that age [58].

#### 3.2.3. Children’s Physical Activity Behavior in Specific Settings

Most of CPARG’s studies have recruited participants through the educational institution attended by the children or adolescents targeted in the study. Typically, the young persons included in a study were observed in the school or childcare center they attended, and the characteristics of those institutions were measured through information gathered from teachers, administrators, parents and/or the children themselves. This information allowed examination of associations between the characteristics of the educational institutions and children’s physical activity. In some cases, activities in which children engaged in settings outside of school were studied. For example, organized youth sport programs are widely available in schools and communities, and these programs are ostensibly important sources of physical activity. In two independent studies, students were followed from middle school into high school, and in both studies it was found that those who reported participation in sports programs were more physically active than their counterparts who did not participate in sports [59,60].

In LEAP, a study of high school girls, it was observed that girls reported more physical activity if they were currently enrolled in physical education classes in school [61]. Furthermore, students who were employed outside of school were more physically active than girls who did not have jobs [62]. Much of CPARG’s work on the influence of settings on physical activity was undertaken with 3–5-year-old children who attended structured childcare centers. These studies found that children’s overall level of participation in moderate-to-vigorous intensity physical activity is low while they are in childcare centers. However, there was considerable variability in children’s activity levels across centers [45], suggesting that differing instructional practices could be important factors. This hypothesis was supported in another study, in an independent sample, showing that certain center and classroom factors were associated with low physical activity [63]. The important influence of childcare center instructional practices was demonstrated in a study showing that children attending Montessori centers were more physically active than counterparts attending traditional centers [64]. Further, it was observed that children’s physical activity levels increase markedly when they transition to an outside space [65].

#### 3.2.4. Children’s Participation in Specific Forms of Physical Activity

Most of CPARG’s studies included instruments that assessed children’s participation in specific modes of physical activity. For older children and youth this information was collected by self-report whereas in younger children it was assessed by direct observation or parental reports. Several distinct study designs were used in exploring children’s participation in specific forms of physical activity. In two studies children who were shown to be highly physically activity via accelerometry were compared to less active children. In a study of preschool-aged children, highly active children were directly observed and found to engage more frequently in running, crawling, climbing, jumping, and skipping [66]. Highly active middle school girls, compared with less active girls, reported more frequent participation in physical activity classes and lessons, and team and individual sports programs [67]. In the same group of middle school girls, age trends were examined, and it was found that in the transition from 6th to 8th grade girls participated in similar specific activities but for longer time periods and more frequently in organized programs [68]. In a comparison of Black and White high school girls, black girls reported more frequent participation in social dance and basketball [62].

The CPARG team has given special attention to walking as a highly prevalent form of physical activity. In children transitioning from 5th to 7th grade, nearly half of students reported recent walking for exercise but less than 20% reported walking for transportation [69]. In a study of middle school girls less than 20% of students reported walking before or after school, suggesting a low prevalence of active transport to school [70]. Indices of neighborhood walkability have been developed by several organizations, and two of these were examined in a CPARG study of walking behavior in children. The indices, which yielded higher walkability scores in urban compared to rural settings, were not significantly associated with self-reported or objective indicators of walking behavior in children residing in either setting [71]. In a laboratory study of girls’ rates of energy expenditure during walking, a very high level of inter-subject variability was observed. For slow walking (2.5 miles per hour) the rate of energy expenditure was 14.0 + 3.7 kJ/min with a range of 8.8 to 23.5 kJ/min. For brisk walking (3.5 miles per hour) energy expenditure was 19.1 + 5.2 kJ with range of 8.6 to 36.7 [35]

In summary, CPARG contributed to a better understanding of physical activity behavior in preschool-aged and school-aged children, how physical activity levels change over time, and how it varies by context and in different forms.

### 3.3. Findings Related to Determinants and Correlates of Physical Activity in Children and Youth

Forty-eight published articles from the six projects reported findings in this category; we used 12 of these papers to develop this section, primarily non-intervention studies featuring longitudinal designs, multiple domains of influence, and reporting primary study aims (see Table 3 for articles cited). The simpler designs with a focus on limited numbers of domains were important work that enabled the research team to develop the more sophisticated studies upon with this section is built. Twenty-seven papers used cross-sectional designs and 21 used longitudinal designs. Outcomes included self-reported physical activity, device-based physical activity, METS (metabolic equivalent for task, a unit that estimates the amount of energy used by the body during physical activity), and cardiorespiratory fitness. Possible determinant domains included social cognitive, social environment, physical environment, behavioral, and race/ethnicity. Eleven papers focused on one determinant domain, most commonly the environment. Sixteen focused on two domains, most commonly elements of both the social and physical environments, and 21 focused on three or more domains.

#### 3.3.1. Correlates of Physical Activity in Preschool-Aged Children

Parent perceptions, parent variables, and childcare environments are related to physical activity in preschool-aged children. Parent perceptions associated with child’s physical activity include child’s athletic competence [72] and perceived importance of child physical activity and child’s enjoyment of physical activity [73]. Family support of child physical activity, a parent characteristic, is associated with child’s physical activity. Parent physical activity was indirectly related to child physical activity through its influence on family support [73].

CPARG studies identified a home characteristic, presence of home physical activity equipment, that is associated with child physical activity in preschool-aged children [73]. Childcare center characteristics associated with child physical activity include higher quality of the childcare center, physical environments with less fixed and more portable playground equipment, lower use of electronic media, and larger playgrounds [74].

#### 3.3.2. Determinants of Physical Activity as Children Transition from Elementary to Middle School

Psychosocial factors, child sports participation, and supportive physical activity environments are associated with physical activity as children transition from elementary to middle school. Behavioral factors that influence physical activity during this transition include sport participation [32,75] and time spent outdoors [32]. Psychosocial factors that influence physical activity during the transition include self-efficacy for overcoming physical activity barriers [75,76], parent support and parent encouragement for physical activity [32,76,77], and friend support for physical activity [76].

Environmental factors that influence physical activity as children transition from elementary to middle school include physical activity equipment in the home [77], favorable neighborhood physical activity environment (no incivilities, presence of social spaces and PA facilities) [77], social spaces for physical activity in the community [32], number of physical activity facilities near the child’s home [32] and school intramural activities [32].

#### 3.3.3. Determinants of Physical Activity as Children Transition from Middle to High School

These papers focused primarily on psychosocial influences and provided some of the earliest evidence that change in students’ beliefs and motives about physical activity were related to change in physical activity in direct, indirect (i.e., mediated), and interactive (i.e., moderated) ways. Within psychosocial influences, perceived behavioral control, social support, barriers self-efficacy, and motivation are important influences on physical activity in the transition from middle to high school. However, these factors may change over time and their effects on physical activity over time are complex and may depend on changes in other psychosocial factors. Furthermore, perceptions of the physical environment may exert indirect (i.e., mediated) influences on physical activity. For example, perceived physical environmental factors indirectly influenced self-reported physical activity via barriers self-efficacy, and perceived social environmental factors (social support) both directly and indirectly (via barriers self-efficacy) influenced self-reported physical activity in this sample of older adolescent girls [78].

Self-efficacy may be largely stable over high school, but its level (high or low) interacts with (i.e., moderates) changes in social support over time [79]. Similarly, higher intrinsic motivation is associated with less decline in physical activity, depending upon changes in physical activity motivational goals (e.g., appearance, social, and competence goals) [80]. These complex relationships among environmental and psychosocial variables are consistent with concepts in social cognitive theory and social ecological models.

#### 3.3.4. Determinants of Physical Activity as Children Transition from Elementary to Middle to High School

Like the results for the transition from middle to high school, the relationships between determinants and physical activity from elementary to high school are complex. For example, when we examined psychosocial variables, neither 5th grade levels of self-efficacy nor change in self-efficacy was directly related to decline in physical activity, but physical activity declined most in students who experienced bigger declines in self-efficacy and maintained higher perceptions of barriers to physical activity, compared to students with higher self-efficacy and lower perceived barriers [81]. Students who experienced bigger declines in self-efficacy concurrent with bigger declines in enjoyment or fitness goals, or smaller declines in appearance or social goals, also experienced greater declines in physical activity [81].

When we examined a comprehensive set of child, parent/home, school, and neighborhood factors [82], variables from child, parent/home, and neighborhood domains exerted consistent main effects on physical activity across the transition from elementary to high school. These included four child variables (self-efficacy, self-schema, sport participation, and weekday outdoor hours); one parent characteristic (belief in importance of child participating in sports and physical activity); and two neighborhood characteristics (safe to play outside and availability of well-maintained physical activity facilities, which indicates the availability of physical activity facilities). The analysis used in this study also identified the effects of time-varying determinants associated with children’s age-related change in physical activity and found variables from multiple domains that were significantly associated with children’s change in physical activity. These variables had more complex relationships with physical activity. For example, for enjoyment motivation, those with higher scores were more physically active at the younger ages, but the difference diminished with increasing age, whereas appearance motivation and the availability of physical activity equipment in the home were increasingly positively associated with physical activity as children aged.

In summary, determinants at the child, parent/home, and neighborhood domains exert important influences on physical activity as children transition from elementary to high school, which indicates the importance of addressing all these factors in policy, program, and environmental interventions in school and community settings.

### 3.4. Findings Related to the Effectiveness of School-Based Interventions to Promote Physical Activity in Children and Youth

CPARG interventions, presented in 20 articles included in the critical review, are Active Winners, LEAP, TAAG (which included both a study-directed intervention and a community-directed intervention), and SHAPES. Manuscripts described the study protocol and/or the intervention, presented in-depth case studies and formative work, and presented intervention outcome results. Additional manuscripts presented factors that mediated or moderated intervention outcomes, featured process evaluation, implementation, or sustainability results, and described dissemination or translation results.

#### 3.4.1. Characteristics all CPARG Interventions Have in Common

All CPARG interventions were conceptually based, included multiple components, and targeted multiple domains of influences on physical activity [83,84,85,86,87,88,89,90].

#### 3.4.2. Characteristics Effective CPARG Interventions (i.e., Those That Changed Physical Activity Behavior) Have in Common

CPARG interventions that had a positive impact on physical activity were LEAP [83,84,91], the TAAG community-directed intervention [85,86], and SHAPES [87,88,89]. Several intervention characteristics and approaches to implementation were common to these effective CPARG interventions.

Effective interventions such as LEAP and SHAPES used public health and environmental approaches to achieve population impact rather than focusing on individual behavior change. These same interventions were designed to be adaptable and were guided by theory and/or evidence-based principles or goals, and they enabled teachers and schools to have some flexibility in choosing strategies, in order to take advantage of local skills and resources and meet local students’ needs. This approach requires that researchers and site-based implementers (e.g., teachers) have a deep understanding of the essential elements of the intervention and the mechanisms through which it produces the desired changes, which can be theory- and/or empirically based. It also requires an understanding of how the school “works” at a detailed level, as well as a focus on a few intervention priorities that are feasible to carry out in the real-world context. Finally, as shown in LEAP and SHAPES, cultivating administrator/organizational support for physical activity is essential.

Forming partnerships with schools, developing good working relationships with stakeholders, and identifying setting-based champions are characteristic of effective intervention approaches, such as those used in LEAP, TAAG community-directed, and SHAPES. Training staff in the intervention setting (school or childcare setting) to implement the intervention requires a significant investment of researcher effort up front, but results in more intervention effects in the long run. For example, LEAP Champions were effective change agents in their schools, and trained SHAPES teachers were effective classroom change agents.

#### 3.4.3. Contributions to Intervention Effectiveness (Looking Inside the “Black Box”)

For an intervention to be effective, it must be implemented as designed and must reach the intended population. Implementation and reach can be monitored by conducting comprehensive process evaluation and implementation monitoring, which allow researchers to make adjustments as the intervention proceeds [84,91,92,93,94,95]. Furthermore, schools with higher levels of intervention implementation had better physical activity results [91], as did schools that maintained intervention practices over time [96]. To understand how the intervention is effective (versus just whether it *is* effective) requires using both quantitative (process evaluation, mediation and moderation analyses) and qualitative (process evaluation and case study) methods. This must be planned and budgeted from the beginning of the project.

Assessing factors that mediate and moderate the intervention is important for understanding how the intervention works. For LEAP, variables that partially mediated the effects of the intervention were enjoyment and self-efficacy [97]. For SHAPES, selected organizational characteristics moderated the effects of the intervention [98]. Conducting qualitative case studies, such as those by Felton et al. for LEAP [99] and Howie et al. for SHAPES [100], provide an in-depth understanding of how flexible and adaptive environmental interventions were carried out in high school and childcare settings, respectively.

#### 3.4.4. Effective Approaches to Conducting Interventions in School Settings

Understanding and working with the setting and context are crucial. Using conceptually based approaches and targeting change efforts at multiple ecological levels is necessary but not sufficient; it is also important to implement the intervention in partnership with stakeholders and in a manner that “fits” the local context and resources. Flexible and adaptive interventions that are well-guided by theory and/or evidence enable local school personnel to carry out the intervention effectively in a way that works for their unique settings. Process evaluation/implementation monitoring is essential for keeping interventions on track and for assessing the impact of level of implementation on outcomes. Study design, measurement, and analysis should be planned from the beginning to enable examination of mediators and moderators of intervention effects.

It is also important to learn from mistakes and challenges in each project and use that information to improve the next project. CPARG study designs, intervention designs, partnerships with stakeholders, measurement, analyses, implementation strategies, and implementation monitoring grew in sophistication over time as we learned from experience. Investigators should plan from the beginning to facilitate and measure sustainability of change, as in LEAP [92,96] and to conduct dissemination and translation research in order to get effective interventions out in the “real world,” as in SHAPES [101,102].

### 3.5. Findings about Race/Ethnicity and Physical Activity in Children and Adolescents

Fifteen articles that were included in the four review areas presented in the sections above were also relevant for the fifth focal CPARG area of race/ethnicity and physical activity. Ten of these were included in one of the previous categories and five were unique to this section.

The racial composition of participants in CPARG projects largely mirrors that of the population of South Carolina, with large proportions of Black participants. For example, Active Winners intervention participants were 88% Black [90], LEAP participants were 49% Black [83], and TRACK participants were 35% Black [32]. This has positioned the CPARG research team to examine the role of physical activity in children and adolescents of different races.

In addition, in the years since CPARG began studying physical activity in children, the Hispanic population in South Carolina has grown. From 2012 to 2022, the percentage of Hispanic/Latino children in PK-12th grade rose from 7.1% to 12.3% (South Carolina Department of Education), which enabled us to include Hispanic/Latino participants in more analyses. Furthermore, the TAAG study was conducted in six field centers (Minnesota, Columbia SC, Baltimore/DC, San Diego, Tucson, and New Orleans), which allowed larger percentages of Hispanic girls to be included in the study (e.g., 20% of sample in Pate et al. 2009) [46].

In CPARG studies, race/ethnicity was reported by trained data collection staff (Active Winners) parents (CHAMPS, SHAPES) or self-reported by the child (LEAP, TAAG, TRACK). The responder chose one or more races/ethnicities that were then categorized into up to four groups, including White, Black, Hispanic, and Other (including multiracial). In this section we critically review 15 articles selected from all research projects.

#### 3.5.1. The Role of Race/Ethnicity for Correlates/Determinants of Physical Activities

Several papers dealt with measurement issues pertaining to correlates/determinants, such as determining group invariance and the applicability of social constructs in both Black and White children. For example, some constructs, such as physical self-description (assessed by the Physical Self Description Questionnaire or PSDQ) [39], work equally well with Black and White girls. However, not all instruments work the same for both groups. For example, Motl et al. determined that, of six subscales for social provisions, Black and White girls had only three in common–Guidance, Nurturance, and Reassurance of Worth [43]. Further, the data suggested that the theory of planned behavior has limited utility among Black adolescent girls [103]. In addition, there are differences between White and Black girls for some constructs. For example, white girls had higher latent mean scores than Black girls on measures of attitude and self-efficacy [38], and several papers found that correlates of physical activity varied by racial/ethnic groups (White, Black and Hispanic) in girls [104,105].

#### 3.5.2. Race/Ethnicity in Types and Amounts of Physical Activities

The studies found differences between Black and White adolescent girls in participation in 11 physical activities and 3 sedentary activities [106], and some found race differences in the amount of physical activity performed. More 5th grade White girls participated in self-reported vigorous physical activity as compared to Black girls, but no differences were found among boys [75]. White girls had higher levels of total self-reported physical activity from 8th to 12th grade, but over time the difference declined [107]. No race differences were found in directly observed physical activity in preschool-aged children [45]. Age-related declines in objectively-measured physical activity were studied in several papers [53,58]. Although declines tended to be larger in black girls from 6th to 8th grade, ethnic differences were not significant [53]. Similarly, age-related decline in total physical activity in boys and girls from 10 to 17 years of age did not differ by race/ethnicity [58].

#### 3.5.3. Race/Ethnicity and Results of Intervention

SHAPES had a significant intervention effect on objectively-measured moderate-to-vigorous intensity physical activity in 4-year-old children even after adjusting for sex, race, and parent education, which indicates that program effectiveness did not vary by race/ethnicity [89]. In the TAAG middle school intervention, no significant interactions between treatment and race/ethnicity were observed for either the primary outcome variable or any of the secondary outcome variables [85]. The LEAP randomized controlled trial effectively increased enjoyment and physical activity among both Black and White high school girls [97]. The complex effects of age, gender, and race/ethnicity on physical activity make it challenging to draw conclusions about race/ethnicity based on a limited number of projects that span childcare settings (SHAPES), middle school (TAAG), and high school (LEAP) youth, some of which focused on girls only (e.g., LEAP and TAAG). CPARG and other research groups continue work to understand these complex relationships. It is clear, however, that correlates of physical activity varied by race/ethnicity, which suggests the need for culturally tailored strategies in physical activity interventions in diverse populations [104,105].

### 3.6. Synthesis across Five Categories

CPARG has focused on understanding physical activity in school-aged youth for 30+ years. Our experience over time with a variety of methods and in different settings with varying populations provides us a “big picture” view of physical activity promotion in children. This is a summary of what we have learned. Helping children become more physically active involves assessing physical activity levels, patterns, specific forms, and contexts; identifying and measuring correlates/determinants of physical activity; and conducting school-based physical activity interventions. Understanding multiple domains of physical activity determinants from both theory and research enables researchers and practitioners to select or design age-appropriate, valid, and reliable instruments to assess determinants of physical activity in programs or populations of children and adolescents. Focusing on the same determinants also enables them to create effective interventions, environments, programs, and policies in school and other settings that promote physical activity for children and adolescents. Physical activity interventions and measurement of determinants must address race/ethnicity differences, ensuring that measurement instruments and intervention strategies are culturally appropriate. School-based physical activity interventions should also include comprehensive process evaluation and implementation monitoring.

It is important to design interventions that can be adapted and are guided by theory and/or evidence-based principles, and that provide teachers and schools with some flexibility to choose specific strategies that take advantage of local skills and resources, as well as local students’ needs. Ideally, interventions to promote physical activity are accomplished in partnership with schools, including working with a setting-based champion, and are based on working within schools’ organizational priorities (e.g., an appreciation for the educational mission of schools).

Change in physical activity and determinants of physical activity as children transition from childhood to adolescence is complex; however, several principles and patterns have emerged in our work.

- Physical activity levels are variable and, overall, most children do not attain recommended levels of physical activity.
  1. School-aged children spend most of their time in sedentary or light intensity physical activity.
  2. About half of children in childcare programs were physically active for less than the recommended 30 min per hour.
- Distinct groups of children exhibit different patterns of physical activity as they age
  1. Physical activity declines in most but not all children over time, with the steepest decline before the age of 14.
  2. Most children tend to remain at a similar ranking within a group over time.
- Children involved in sports, girls enrolled in physical education, and girls employed outside of school were more physically active than their counterparts.
- Walking for exercise is more common than walking for transportation.
  1. Walking behavior varies considerably in intensity among youth.
  2. Though walkability index scores varied by urban and rural settings, they were not related to walking behavior in either setting.
- Overall, participation in moderate-to-vigorous physical activity is low in children attending childcare centers.
  1. There is much center-to-center variability suggesting the importance of instructional practices.
  2. Physical activity levels increase when children are outside.
- Influences on physical activity in youth include factors from all domains: the child level, the social environment, and the physical environment at the home, neighborhood, community, and school levels. For example:
  1. For preschool-aged children, parent perceptions of child athletic competence, parent support, presence of home physical activity equipment, and larger childcare center playgrounds with less fixed and more portable equipment are related to child physical activity.
  2. For elementary to middle school children, child sports participation, time outdoors, barriers self-efficacy, parent and friend support for physical activity, and supportive neighborhood environments with social spaces and equipment are related to physical activity.
  3. Important psychosocial influences as children transition from middle to high school include perceived behavior control, social support, barriers self-efficacy, and motivation, along with perceptions of the environment. These influences may affect physical activity directly and indirectly (through mediation) and may interact (through moderation) with each other over time.
  4. Important influences on physical activity as children transition from elementary to middle to high school come from multiple domains, including the child, parent/home, and neighborhood environment. These include self-efficacy, self-schema, enjoyment motivation, appearance motivation, sport participation, weekday outdoor hours, importance of child participating in sports and physical activity, safe to play outside, and the availability and quality of physical activity facilities.
- Additional research examining the role of race/ethnicity in physical activity behavior, conceptualizing and measuring physical activity correlates/determinants, and in the design of physical activity interventions is needed.
  1. In our studies, Black and White girls reported different physical activities, different theoretical constructs seemed to be applicable to different races/ethnicities, and correlates of physical activity varied by race/ethnicity, which suggests the need for additional research upon which to develop culturally tailored strategies in physical activity interventions in diverse populations.
  2. Nevertheless, positive outcomes from several large trials conducted by CPARG were independent of race/ethnicity.

A major strength of this article’s approach is that it considers the findings of multiple articles that used varying methodologies applied in different settings and samples of children. While the findings of a specific analysis may be well known (i.e., well-cited in the literature), the overall impact of individual articles may be diffused over an extended timeframe and the broad range of scientific outlets across multiple disciplines. Similarly, patterns of findings over several decades became more evident with a focused synthesis. Accordingly, this synthesis provides an understanding of physical activity promotion in children that is deeper and broader than the impact of previously published individual articles.

## 4. Conclusions

Over the past 30 years, CPARG has contributed a body of work dedicated to understanding and ultimately improving physical activity levels in young children, school-aged children, and adolescents, with the ultimate goal of enabling more youth to realize the physical, mental, social, and academic benefits of regular physical activity. This endeavor required:

- Developing and refining effective measures of physical activity and physical activity determinants.
- Developing an understanding of physical activity behavior in youth of all ages and how it changes over time and by setting.
- Utilizing knowledge of multiple domains of influences on physical activity, including understanding how:
  1. Social environments and natural and built physical environments at home, in school, in the neighborhood, and in the community affect physical activity.
  2. Social cognitive characteristics of the child influence physical activity.
- Developing effective partnerships with schools to carry out flexible physical activity interventions based on determinants of physical activity.
- Developing an understanding of the role of race/ethnicity in physical activity of children and adolescents.

This manuscript focused on CPARG’s completed projects that are most directly relevant to school settings. As previously noted, past and current CPARG projects also examine home and community settings, which were beyond the scope of this paper. For example, LAUNCH (Linking Activity, Nutrition, and Child Health) is a current and ongoing observational study to examine the relationships among physical activity, nutrition, and weight status in children as they develop from infancy to preschool age. CPARG continues to assess physical activity, examine influences on physical activity, and promote physical activity across the early lifespan. Past, present, and future CPARG efforts will facilitate the development of interventions, environments, programs, and policies to promote physical activity in youth, with the goal of improving population health and well-being.

## Figures and Tables

**Table 1:** Overview of Selected CPARG Research Projects Featured in This Manuscript.

Project	Funding Timeframe	Design and Sample	Methods	Key Findings
Active Winners	1993–1998	Quasi-experimental examination of effects of an afterschool and summer physical activity program in one rural SC community compared to a control community	Intervention delivery was over 19 months from summer 1994 following the 5th grade (*n* = 558) to midway through the 7th grade. Data were collected at baseline, mid-point in the intervention period, and follow-up.	There were no significant intervention effects. Process evaluation revealed high fidelity but limited reach and completeness.
LEAP	1997–2007	Group randomized examination of the effects of a high school physical activity intervention for girls in 12 intervention and 12 SC high schools	Intervention was delivered to girls in the 9th grade (*n* = 2744). Baseline testing was performed during the spring of the student’s 8th grade year with follow-up administered one and two years later.	Intervention group engaged in significantly more vigorous physical activity. Data suggest that this effect was not explained solely by participation in physical education.
TAAG	2005–2008	Randomized, multi-center field trial (one site out of 6 field sites) of an intervention to promote physical activity in middle school girls, conducted in 36 middle schools (6 schools per site)	The primary, research staff-directed intervention took place over 2 years. MVPA was assessed in cross-sectional samples of 6th (*n* = 1721)and 8th (*n* = 3504) grade girls taken from each school approximately 18 months apart. In the 3rd year, community personnel directed intervention delivery (*n* = 3502).	There were no significant intervention effects in the 2-year, staff-directed intervention, but in the 3rd-year program, girls in the intervention schools were more physically active compared to the control schools.
CHAMPS	2003–2008	Cross-sectional, observational study to describe associations between MVPA and the factors that are hypothesized to influence MVPA	Conducted in 24 licensed childcare centers with 720 children ages 3 to 5.	CHAMPS enabled the team to develop measures, to examine motor skills, physical activity, and influences on physical activity in preschool-aged children, and to examine the importance of policy and environment in childcare settings.
SHAPES	2008–2012, 2013–2016, and 2017–2019	A sequence of three studies: a randomized trial of 16 childcare centers (8 intervention and 8 control classrooms of four-year-olds), followed by translating SHAPES to an online format, and then state-wide dissemination of the online program	Intervention childcare centers implemented SHAPES in their classrooms (369 children) over a three-year period. The SHAPES intervention and training were translated to online delivery and tested, followed by statewide dissemination.	Children in intervention childcare centers engaged in significantly more MVPA than those in control childcare centers. The change to online delivery was characterized by high levels of implementation, and statewide dissemination had comparable implementa-tion completeness and fidelity.
TRACK	2008–2017	Observational longitudinal cohort study to examine physical activity and the influences of multiple domains of influences on physical activity.	Over 1100 5th graders were recruited to participate in the study. Participants completed measures during their 5th, 6th, 7th, 9th, and 11th grade years	TRACK enabled the team to track youth physical activity and examine multiple domains of influences on physical activity over the transitions from elementary school to middle school to high school.

Note: MVPA = moderate-to-vigorous physical activity.

**Table 2:** Guiding Questions for Critical Review of Selected CPARG Articles.

**Measurement of Physical Activity and Determinants** What contributions has CPARG made to the: Measurement of physical activity in preschool-aged children? Measurement of physical activity in school-aged children? Measurement of correlates/determinants of physical activity in school-aged children?
**Physical Activity Behavior** What have we learned about: Typical physical activity levels, and compliance with physical activity guidelines? Age-related change in physical activity and tracking? Children’s physical activity behavior in specific settings? Children’s participation in specific forms for physical activity?
**Correlates/Determinants of Physical Activity** What have we learned about the: Correlates of physical activity in preschool-aged children? Determinants of physical activity as children transition from elementary to middle school? Determinants of physical activity as children transition from middle to high school? Determinants of physical activity as children transition from elementary to middle to high school?
**Physical Activity Interventions** What characteristics do: All CPARG interventions have in common? Effective CPARG interventions (i.e., those that changed PA behavior) have in common? What contributes to intervention effectiveness (looking inside the “black box”)? What do we conclude about effective approaches to conducting interventions in school settings?
**Race/Ethnicity and Physical Activity** What have we learned about: The role of race/ethnicity for correlates/determinants of physical activities? Race/ethnicity in types and amounts of physical activities? Race/ethnicity and results of interventions?

**Table 3:** Articles Referenced in Each Review Section by Project.

Study	Reference Numbers for Articles Included in Five Review Sections				
	Measurement of Physical Activity and Determinants	Physical Activity Behavior	Determinants/Correlates of Physical Activity	School-Based Physical Activity Interventions	Race/Ethnicity and Physical Activity
Active Winners	26, 36	51	75	90	75
LEAP	27, 28, 38, 39, 40, 42, 43, 44	52, 59, 61, 62	78	83, 84, 91, 92, 96, 97, 99	38, 39, 43, 97, 103, 106, 107
TAAG	33, 35, 41	46, 53, 54, 67, 68, 70	-	85, 86	53, 85,104
CHAMPS	14, 15, 16, 20, 21, 22, 23, 24, 25	45, 50, 65, 66	72, 73, 74		45
SHAPES	-	63, 64	-	87, 88, 89, 93, 94, 95, 98, 100, 101, 102	89
TRACK	34, 37	47, 48, 49, 55, 56, 57, 58, 60, 69, 71	32, 76, 77, 79, 80, 81, 82	-	58, 105

## Supplementary Materials

The following are available online at https://www.mdpi.com/article/10.3390/ijerph192114136/s1, File S1: FCPARG Published Articles Listed by Research Project.

## References

[1] Physical Activity Guidelines Advisory Committee (2018). 2018 Physical Activity Guidelines Advisory Committee Scientific Report.

[2] U.S. Department of Health and Human Services (2018). Physical Activity Guidelines for Americans.

[3] Merlo C.L., Jones S.E., Michael S.L., Chen T.J., Sliwa S.A., Lee S.H., Brener N.D., Lee S.M., Park S. (2020). Dietary and Physical Activity Behaviors Among High School Students—Youth Risk Behavior Survey, United States, 2019. MMWR Suppl..

[4] Nader P.R., Bradley R.H., Houts R., McRitchie S.L., O’Brien M. (2008). Moderate-to-Vigorous Physical Activity from Ages 9 to 15 Years. JAMA.

[5] Troiano R.P., Berrigan D., Dodd K.W., Masse L.C., Tilert T., McDowell M. (2008). Physical activity in the United States measured by ac-celerometer. Med. Sci. Sports Exerc..

[6] Farooq A., Martin A., Janssen X., Wilson M.G., Gibson A.M., Hughes A., Reilly J.J. (2020). Longitudinal changes in moderate-to-vigorous-intensity physical activity in children and adolescents: A systematic review and meta-analysis. Obes. Rev..

[7] Centers for Disease Control and Prevention (2013). Comprehensive School Physical Activity Programs: A Guide for Schools.

[8] Pate R.R., Lau E.Y., Flynn J.I., McDonald S.M., Clennin M.N., Schenkelberg M.A. (2015). Scientific contributions of the Children’s Physical Activity Research Group. J. South Carol. Acad. Sci..

[9] Children’s Physical Activity Research Group. https://sc.edu/study/colleges_schools/public_health/research/research_centers/usc_cparg/.

[10] Arteaga S.S., Loria C.M., Crawford P.B., Fawcett S.B., Fishbein H.A., Gregoriou M., John L.V., Kelley M., Pate R.R., Ritchie L.D. (2015). The Healthy Communities Study: Its Rationale, Aims, and Approach. Am. J. Prev. Med..

[11] Pate R.R., Clennin M., Ms E.R.S., Reed J.A., Dowda M. (2020). Poverty Status Moderates the Relationship between Cardiorespiratory Fitness and Academic Achievement. J. Sch. Health.

[12] Pate R.R., Frongillo E.A., Cordan K., Dowda M., McLain A.C., Torres M.E., Brown W.H., Bucko A., Shull E.R. (2020). Linking Activity, Nutrition, and Child Health (LAUNCH): Protocol for a longitudinal cohort study of children as they develop from infancy to preschool age. BMC Public Health.

[13] Omura J.D., Whitfield G.P., Chen T.J., Hyde E.T., Ussery E.N., Watson K.B., Carlson S.A. (2021). Surveillance of Physical Activity and Sedentary Behavior Among Youth and Adults in the United States: History and Opportunities. J. Phys. Act. Health.

[14] Brown W.H., Pfeiffer K.A., McIver K.L., Dowda M., Almeida J.M., Pate R.R. (2006). Assessing preschool children’s physical activity: The Observational System for Recording Physical Activity in Children-Preschool Version (OSRAC-P). Res. Q. Exerc. Sport.

[15] McIver K.L., Brown W.H., Pfeiffer K.A., Dowda M., Pate R.R. (2009). Assessing children’s physical activity in their homes: The Observa-tional System for Recording Physical Activity in Children-Home. J. Appl. Behav. Anal..

[16] McIver K.L., Brown W.H., Pfeiffer K.A., Dowda M., Pate R.R. (2016). Development and Testing of the Observational System for Recording Physical Activity in Children: Elementary School. Res. Q. Exerc. Sport.

[17] Puhl J., Greaves K., Hoyt M., Baranowski T. (1990). Children’s Activity Rating Scale (CARS): Description and calibration. Res. Q. Exerc. Sport.

[18] Schenkelberg M.A., Brown W.H., McIver K.L., Pate R.R. (2021). An observation system to assess physical activity of children with de-velopmental disabilities and delays in preschool. Disabil. Health J..

[19] Cohen A., McDonald S., McIver K., Pate R., Trost S. (2014). Assessing Physical Activity during Youth Sport: The Observational System for Recording Activity in Children: Youth Sports. Pediatr. Exerc. Sci..

[20] Pate R.R., Almeida M.J., McIver K.L., Pfeiffer K.A., Dowda M. (2006). Validation and Calibration of an Accelerometer in Preschool Children. Obesity.

[21] Pfeiffer K.A., Mciver K.L., Dowda M., Almeida M.J.C.A., Pate R.R. (2006). Validation and calibration of the Actical accelerometer in pre-school children. Med. Sci. Sports Exerc..

[22] Addy C.L., Trilk J.L., Dowda M., Byun W., Pate R.R. (2014). Assessing preschool children’s physical activity: How many days of accel-erometry measurement?. Pediatr. Exerc. Sci..

[23] Byun W., Beets M.W., Pate R.R. (2015). Sedentary Behavior in Preschoolers: How Many Days of Accelerometer Monitoring Is Needed?. Int. J. Environ. Res. Public Health.

[24] Bornstein D.B., Beets M.W., Byun W., Welk G., Bottai M., Dowda M., Pate R. (2011). Equating accelerometer estimates of moderate-to-vigorous physical activity: In search of the Rosetta Stone. J. Sci. Med. Sport.

[25] Beets M.W., Bornstein D., Dowda M., Pate R.R. (2011). Compliance with National Guidelines for Physical Activity in U.S. Preschoolers: Measurement and Interpretation. Pediatrics.

[26] Weston A.T., Petosa R., Pate R.R. (1997). Validation of an instrument for measurement of physical activity in youth. Med. Sci. Sports Exerc..

[27] Pate R.R., Ross R., Dowda M., Trost S.G., Sirard J.R. (2003). Validation of a three-day physical activity recall instrument in female youth. Pediatr. Exerc. Sci..

[28] Motl R.W., Dishman R.K., Dowda M., Pate R.R. (2004). Factorial Validity and Invariance of a Self-Report Measure of Physical Activity among Adolescent Girls. Res. Q. Exerc. Sport.

[29] Argiropoulou E.C., Michalopoulou M., Aggeloussis N., Avgerinos A. (2004). Validity and reliability of physical activity measures in greek high school age children. J. Sports Sci. Med..

[30] Lee K.S., Trost S.G. (2005). Validity and reliability of the 3-Day Physical Activity Recall in Singaporean adolescents. Res. Q. Exerc. Sport.

[31] Trost S.G., Pate R.R., Sallis J.F., Freedson P.S., Taylor W.C., Dowda M., Sirard J. (2002). Age and gender differences in objectively measured physical activity in youth. Med. Sci. Sports Exerc..

[32] Pate R.R., Dowda M., Dishman R.K., Colabianchi N., Saunders R.P., McIver K.L. (2019). Change in Children’s Physical Activity: Predictors in the Transition from Elementary to Middle School. Am. J. Prev. Med..

[33] Treuth M.S., Schmitz K., Catellier D.J., McMurray R.G., Murray D.M., Almeida M.J., Going S., Norman J.E., Pate R. (2004). Defining accelerometer thresholds for activity intensities in adolescent girls. Med. Sci. Sports Exerc..

[34] Dowda M., Dishman R.K., Saunders R.P., Pate R.R. (2021). Associations between three measures of physical activity and selected influ-ences on physical activity in youth transitioning from elementary to middle school. Sports Med. Health Sci..

[35] Pfeiffer K.A., Schmitz K.H., McMurray R.G., Treuth M.S., Murray D.M., Pate R.R. (2006). Physical Activities in Adolescent Girls: Variability in Energy Expenditure. Am. J. Prev. Med..

[36] Saunders R.P., Pate R.R., Felton G., Dowda M., Weinrich M.C., Ward D.S., Parsons M.A., Baranowski T. (1997). Development of questionnaires to measure psychosocial influences on children’s physical activity. Prev. Med..

[37] Dishman R.K., Saunders R.P., McIver K.L., Dowda M., Pate R.R. (2013). Construct Validity of Selected Measures of Physical Activity Beliefs and Motives in Fifth and Sixth Grade Boys and Girls. J. Pediatr. Psychol..

[38] Dishman R.K., Motl R.W., Saunders R.P., Dowda M., Felton G., Ward D.S., Pate R.R. (2002). Factorial invariance and latent mean structure of questionnaires measuring social-cognitive determinants of physical activity among black and white adolescent girls. Prev. Med..

[39] Dishman R.K., Hales D.P., Almeida M.J., Pfeiffer K.A., Dowda M., Pate R.R. (2006). Factorial validity and invariance of the Physical Self-Description Questionnaire among black and white adolescent girls. Ethn. Dis..

[40] Motl R.W., Dishman R.K., Saunders R., Dowda M., Felton G., Pate R.R. (2001). Measuring enjoyment of physical activity in adolescent girls. Am. J. Prev. Med..

[41] Dishman R.K., Motl R.W., Sallis J.F., Dunn A.L., Birnbaum A.S., Welk G.J., Bedimo-Rung A.L., Voorhees C.C., Jobe J.B. (2005). Self-Management Strategies Mediate Self-Efficacy and Physical Activity. Am. J. Prev. Med..

[42] Motl R.W., Dishman R.K., Trost S.G., Saunders R.P., Dowda M., Felton G., Ward D.S., Pate R.R. (2000). Factorial validity and invariance of questionnaires measuring social-cognitive deter-minants of physical activity among adolescent girls. Prev. Med..

[43] Motl R.W., Dishman R.K., Saunders R.P., Dowda M., Pate R.R. (2004). Measuring Social Provisions for Physical Activity among Adolescent Black and White Girls. Educ. Psychol. Meas..

[44] Motl R.W., Dishman R.K., Felton G., Pate R.R. (2003). Self-Motivation and Physical Activity among Black and White Adolescent Girls. Med. Sci. Sports Exerc..

[45] Pate R.R., McIver K., Dowda M., Brown W.H., Addy C. (2008). Directly Observed Physical Activity Levels in Preschool Children. J. Sch. Health.

[46] Pate R.R., Stevens J., Pratt C., Sallis J.F., Schmitz K., Webber L.S., Welk G., Young D.R. (2006). Objectively Measured Physical Activity in Sixth-Grade Girls. Arch. Pediatr. Adolesc. Med..

[47] Lau E.Y., Barr-Anderson D.J., Dowda M., Forthofer M., Saunders R.P., Pate R.R. (2015). Associations between Home Environment and after-School Physical Activity and Sedentary Time among 6th Grade Children. Pediatr. Exerc. Sci..

[48] Dowda M., Ross S.E.T., McIver K.L., Dishman R.K., Pate R.R. (2017). Physical Activity and Changes in Adiposity in the Transition from Elementary to Middle School. Child. Obes..

[49] Lau E.Y., Dowda M., McIver K.L., Pate R.R. (2017). Changes in Physical Activity in the School, Afterschool, and Evening Periods during the Transition from Elementary to Middle School. J. Sch. Health.

[50] Pate R.R., O’Neill J.R., Brown W.H., Pfeiffer K.A., Dowda M., Addy C.L. (2015). Prevalence of Compliance with a New Physical Activity Guideline for Preschool-Age Children. Child Obes..

[51] Pate R.R., Trost S.G., Dowda M., Ott A.E., Ward D.S., Saunders R., Felton G. (1999). Tracking of Physical Activity, Physical Inactivity, and Health-Related Physical Fitness in Rural Youth. Pediatr. Exerc. Sci..

[52] Pate R.R., Dowda M., O’Neill J.R., Ward D.S. (2007). Change in Physical Activity Participation Among Adolescent Girls from 8th to 12th Grade. J. Phys. Act. Health.

[53] Pate R.R., Stevens J., Webber L.S., Dowda M., Murray D.M., Young D.R., Going S. (2009). Age-Related Change in Physical Activity in Adolescent Girls. J. Adolesc. Health.

[54] Trilk J.L., Pate R.R., Pfeiffer K.A., Dowda M., Addy C.L., Ribisl K.M., Neumark-Sztainer D., Lytle L.A. (2012). A Cluster Analysis of Physical Activity and Sedentary Behavior Patterns in Middle School Girls. J. Adolesc. Health.

[55] Pate R.R., Dowda M., Dishman R.K., Gorab J., Bucko A., Saunders R.P. (2022). Longitudinal association of biological maturation with physical activity behaviors in girls transitioning from 5th to 7th grade. Am. J. Hum. Biol..

[56] Taverno Ross S.E., Dowda M., Dishman R.K., Pate R.R. (2016). Classes of Physical Activity and Sedentary Behavior in 5th Grade Children. Am. J. Health Behav..

[57] Pate R.R., Schenkelberg M.A., Dowda M., McIver K.L. (2019). Group-based physical activity trajectories in children transitioning from elementary to high school. BMC Public Health.

[58] Pate R.R., Saunders R.P., Ross S.E.T., Dowda M. (2022). Patterns of age-related change in physical activity during the transition from elementary to high school. Prev. Med. Rep..

[59] Pfeiffer K.A., Dowda M., Dishman R.K., McIver K.L., Sirard J.R., Ward D.S., Pate R.R. (2006). Sport Participation and Physical Activity in Adolescent Females across a Four-Year Period. J. Adolesc. Health.

[60] Shull E., Dowda M., Saunders R.P., McIver K., Pate R.R. (2019). Sport participation, physical activity and sedentary behavior in the transition from middle school to high school. J. Sci. Med. Sport.

[61] Pate R.R., Ward D.S., O’Neill J.R., Dowda M. (2007). Enrollment in physical education is associated with overall physical activity in ad-olescent girls. Res. Q. Exerc. Sport.

[62] Dowda M., Pfeiffer K.A., Dishman R.K., Pate R.R. (2007). Associations among Physical Activity, Health Indicators, and Employment in 12th Grade Girls. J. Women’s Health.

[63] O’Neill J.R., Pfeiffer K.A., Dowda M., Pate R.R. (2016). In-school and Out-of-school Physical Activity in Preschool Children. J. Phys. Act. Health.

[64] Pate R.R., O’Neill J.R., Byun W., McIver K.L., Dowda M., Brown W.H. (2014). Physical activity in preschool children: Comparison between Montessori and traditional preschools. J. Sch. Health.

[65] Pate R.R., Dowda M., Brown W.H., Mitchell J., Addy C. (2013). Physical Activity in Preschool Children with the Transition to Outdoors. J. Phys. Act. Health.

[66] Howie E.K., Brown W.H., Dowda M., McIver K.L., Pate R.R. (2012). Physical activity behaviours of highly active preschoolers. Pediatr. Obes..

[67] Ross S.E.T., Dowda M., Beets M.W., Pate R.R. (2013). Physical activity behavior and related characteristics of highly active eighth-grade girls. J. Adolesc. Health.

[68] Pate R.R., Sallis J.F., Ward D.S., Stevens J., Dowda M., Welk G.J., Young D.R., Jobe J.B., Strikmiller P.K. (2010). Age-Related Changes in Types and Contexts of Physical Activity in Middle School Girls. Am. J. Prev. Med..

[69] Ross S.E.T., Clennin M.N., Dowda M., Colabianchi N., Pate R.R. (2018). Stepping It Up: Walking Behaviors in Children Transitioning from 5th to 7th Grade. Int. J. Environ. Res. Public Health.

[70] Saksvig B.I., Catellier D.J., Pfeiffer K., Schmitz K., Conway T., Going S., Ward D.S., Strikmiller P., Treuth M.S. (2007). Travel by Walking before and after School and Physical Activity among Adolescent Girls. Arch. Pediatr. Adolesc. Med..

[71] Bucko A.G., Porter D.E., Saunders R., Shirley L., Dowda M., Pate R.R. (2021). Walkability indices and children’s walking behavior in rural vs. urban areas. Health Place.

[72] Pfeiffer K.A., Dowda M., McIver K.L., Pate R.R. (2009). Factors Related to Objectively Measured Physical Activity in Preschool Children. Pediatr. Exerc. Sci..

[73] Dowda M., Pfeiffer K.A., Brown W.H., Mitchell J.A., Byun W., Pate R.R. (2011). Parental and Environmental Correlates of Physical Activity of Children Attending Preschool. Arch. Pediatr. Adolesc. Med..

[74] Dowda M., Brown W.H., McIver K.L., Pfeiffer K.A., O’Neill J.R., Addy C.L., Pate R.R. (2009). Policies and Characteristics of the Preschool Environment and Physical Activity of Young Children. Pediatrics.

[75] Trost S.G., Pate R.R., Saunders R., Ward D.S., Dowda M., Felton G. (1997). A Prospective Study of the Determinants of Physical Activity in Rural Fifth-Grade Children. Prev. Med..

[76] Dishman R.K., Dowda M., McIver K.L., Saunders R.P., Pate R.R. (2017). Naturally-occurring changes in social-cognitive factors modify change in physical activity during early adolescence. PLoS ONE.

[77] Colabianchi N., Clennin M.N., Dowda M., McIver K.L., Dishman R.K., Porter D.E., Pate R.R. (2019). Moderating effect of the neighbourhood physical activity environment on the relation between psychosocial factors and physical activity in children: A longitudinal study. J. Epidemiol. Community Health.

[78] Motl R.W., Dishman R.K., Ward D.S., Saunders R.P., Dowda M., Felton G., Pate R.R. (2005). Perceived physical environment and physical activity across one year among adolescent girls: Self-efficacy as a possible mediator?. J. Adolesc. Health.

[79] Dishman R.K., Saunders R.P., Motl R.W., Dowda D.M., Pate R.R. (2008). Self-Efficacy Moderates the Relation between Declines in Physical Activity and Perceived Social Support in High School Girls. J. Pediatr. Psychol..

[80] Dishman R.K., Mciver K.L., Dowda M., Pate R.R. (2018). Declining Physical Activity and Motivation from Middle School to High School. Med. Sci. Sports Exerc..

[81] Dishman R.K., McIver K.L., Dowda M., Saunders R.P., Pate R.R. (2019). Self-efficacy, beliefs, and goals: Moderation of declining physical activity during adolescence. Health Psychol..

[82] Pate R.R., Dowda M., Dishman R.K., Saunders R.P., Cordan K.L., Shull E.R., Bucko A.G., Colabianchi N. (2022). Determinants of change in physical activity in children during the transition from elementary to high school.

[83] Pate R.R., Ward D.S., Saunders R.P., Felton G., Dishman R.K., Dowda M. (2005). Promotion of Physical Activity among High-School Girls: A Randomized Controlled Trial. Am. J. Public Health.

[84] Ward D., Saunders R., Felton G., Williams E., Epping J., Pate R. (2006). Implementation of a school environment intervention to increase physical activity in high school girls. Health Educ. Res..

[85] Webber L.S., Catellier D.J., Lytle L.A., Murray D., Pratt C.A., Young D.R., Elder J.P., Lohman T.G., Stevens J., Jobe J.B. (2008). Promoting Physical Activity in Middle School Girls: Trial of Activity for Adolescent Girls. Am. J. Prev. Med..

[86] Elder J.P., Lytle L., Sallis J.F., Young D.R., Steckler A., Simons-Morton D., Stone E., Jobe J.B., Stevens J., Lohman T. (2006). A description of the social-ecological framework used in the trial of activity for adolescent girls (TAAG). Health Educ. Res..

[87] Pfeiffer K.A., Saunders R.P., Brown W.H., Dowda M., Addy C.L., Pate R.R. (2013). Study of Health and Activity in Preschool Environments (SHAPES): Study protocol for a randomized trial evaluating a multi-component physical activity intervention in preschool children. BMC Public Health.

[88] Howie E.K., Brewer A., Brown W.H., Pfeiffer K.A., Saunders R.P., Pate R.R. (2014). The 3-year evolution of a preschool physical activity intervention through a collaborative partnership between research interventionists and preschool teachers. Health Educ. Res..

[89] Pate R.R., Brown W.H., Pfeiffer K.A., Howie E.K., Saunders R.P., Addy C.L., Dowda M. (2016). An Intervention to Increase Physical Activity in Children: A Randomized Controlled Trial with 4-Year-Olds in Preschools. Am. J. Prev. Med..

[90] Pate R.R., Saunders R.P., Ward D.S., Felton G., Trost S.G., Dowda M. (2003). Evaluation of a community-based intervention to promote physical activity in youth: Lessons from Active Winners. Am. J. Health Promot..

[91] Saunders R.P., Ward D., Felton G.M., Dowda M., Pate R.R. (2006). Examining the link between program implementation and behavior outcomes in the lifestyle education for activity program (LEAP). Eval. Program Plan..

[92] Saunders R.P., Pate R.R., Dowda M., Ward D.S., Epping J.N., Dishman R.K. (2011). Assessing sustainability of Lifestyle Education for Activity Program (LEAP). Health Educ. Res..

[93] Lau E.Y., Saunders R.P., Beets M.W., Cai B., Pate R.R. (2017). Factors influencing implementation of a preschool-based physical activity intervention. Health Educ. Res..

[94] Kennedy A.B., Schenkelberg M., Moyer C., Pate R., Saunders R.P. (2017). Process evaluation of a preschool physical activity intervention using web-based delivery. Eval. Program Plan..

[95] Saunders R.P., Pfeiffer K., Brown W.H., Howie E.K., Dowda M., O’Neill J.R., McIver K., Pate R.R. (2017). Evaluating and Refining the Conceptual Model Used in the Study of Health and Activity in Preschool Environments (SHAPES) Intervention. Health Educ. Behav..

[96] Pate R.R., Saunders R., Dishman R.K., Addy C., Dowda M., Ward D.S. (2007). Long-Term Effects of a Physical Activity Intervention in High School Girls. Am. J. Prev. Med..

[97] Dishman R.K., Motl R.W., Saunders R., Felton G., Ward D.S., Dowda M., Pate R.R. (2005). Enjoyment Mediates Effects of a School-Based Physical-Activity Intervention. Med. Sci. Sports Exerc..

[98] Saunders R.P., Schenkelberg M.A., Moyer C., Howie E.K., Brown W.H., Pate R.R. (2019). The translation of an evidence-based preschool physical activity intervention from in-person to online delivery of professional development to preschool teachers. Transl. Behav. Med..

[99] Felton G., Saunders R.P., Ward D.S., Dishman R.K., Dowda M., Pate R.R. (2005). Promoting physical activity in girls: A case study of one school’s success. J. Sch. Health.

[100] Howie E.K., Brewer A.E., Dowda M., McIver K.L., Saunders R.P., Pate R.R. (2015). A Tale of 2 Teachers: A Preschool Physical Activity Intervention Case Study. J. Sch. Health.

[101] Howie E.K., Brewer A.E., Brown W.H., Saunders R.P., Pate R.R. (2016). Systematic dissemination of a preschool physical activity inter-vention to the control preschools. Eval. Program Plan..

[102] Saunders R.P., Dowda M., Pfeiffer K.A., Brown W.H., Pate R.R. (2019). Childcare Center Characteristics Moderate the Effects of a Physical Activity Intervention. Int. J. Environ. Res. Public Health.

[103] Trost S.G., Pate R.R., Dowda M., Ward D.S., Felton G., Saunders R. (2002). Psychosocial correlates of physical activity in white and Afri-can-American girls. J. Adolesc. Health.

[104] Kelly E.B., Parra-Medina D., Pfeiffer K.A., Dowda M., Conway T.L., Webber L.S., Jobe J.B., Going S., Pate R.R. (2010). Correlates of Physical Activity in Black, Hispanic, and White Middle School Girls. J. Phys. Act. Health.

[105] Barr-Anderson D.J., Flynn J.I., Dowda M., Ross S.E.T., Schenkelberg M.A., Reid L.A., Pate R.R. (2017). The Modifying Effects of Race/Ethnicity and Socioeconomic Status on the Change in Physical Activity from Elementary to Middle School. J. Adolesc. Health.

[106] Dowda M., Pate R.R., Felton G.M., Saunders R., Ward D.S., Dishman R.K., Trost S.G. (2004). Physical Activities and Sedentary Pursuits in African American and Caucasian Girls. Res. Q. Exerc. Sport.

[107] Dowda M., Dishman R.K., Pfeiffer K.A., Pate R.R. (2007). Family support for physical activity in girls from 8th to 12th grade in South Carolina. Prev. Med..

