# Supplementary material for: A Summary of One Research Team’s Contributions to Understanding Physical Activity Behavior in Children and Youth
Source: Int J Environ Res Public Health. 2022 Oct 29;19(21):14136. doi: 10.3390/ijerph192114136 (PMC9655861; doi:10.3390/ijerph192114136)
Supplement: Supplementary file 1 [file ijerph-19-14136-s001.zip › ijerph-1984681-supplementary.pdf]

Supplemental File S1: CPARG Published Articles Listed by Research Project

Active Winners Articles

Felton, G.; Parsons, M. A.; Ward, D. S.; Pate, R. R.; Saunders, R. P.; Dowda, M.; Trost, S. Tracking of Avoidance of Alcohol Use and Smoking Behavior in a Fifth Grade Cohort over Three Years. *Public Health Nursing* **1999**, *16(1),32-40.*

Felton, G. M.; Parsons, M. A.; Pate, R. R.; Ward, D.; Saunders, R.; Valois, R.; Dowda, M.; Trost, S. Predictors of Alcohol Use among Rural Adolescents. *The Journal of Rural Health* **1996**, *12(5), 378-385.*

Felton, G. M.; Pate, R. R.; Parsons, M. A.; Ward, D. S.; Saunders, R. P.; Trost, S.; Dowda, M. Health Risk Behaviors of Rural Sixth Graders. *Research in Nursing & Health* **1998**, *21(6), 475-485.*

Pate, R. R.; Saunders, R. P.; Ward, D. S.; Felton, G.; Trost, S. G.; Dowda, M. Evaluation of a Community-Based Intervention to Promote Physical Activity in Youth: Lessons from Active Winners. *Am J Health Promot* **2003**, *17*(3), 171–182.

Pate, R. R.; Trost, S. G.; Dowda, M.; Ott, A. E.; Ward, D. S.; Saunders, R.; Felton, G. Tracking of Physical Activity, Physical Inactivity, and Health-Related Physical Fitness in Rural Youth. *Pediatric Exercise Science* **1999**, *11*(4), 364–376.

Pate, R. R.; Trost, S. G.; Felton, G. M.; Ward, D. S.; Dowda, M.; Saunders, R. Correlates of Physical Activity Behavior in Rural Youth. *Research Quarterly for Exercise and Sport* **1997**, *68(3), 241-248.*

Saunders, R. P.; Pate, R. R.; Felton, G.; Dowda, M.; Weinrich, M. C.; Ward, D. S.; Parsons, M. A.; Baranowski, T. Development of questionnaires to measure psychosocial influences on children's physical activity. *Preventive Medicine* **1997**, *26(2), 241-247.*

Trost, S. G.; Morgan, A. M.; Saunders, R.; Felton, G.; Ward, D. S.; Pate, R. R. Children's Understanding of the Concept of Physical Activity. *Pediatric Exercise Sciences* **2000**, *12* (3), 293–299.

Trost, S. G.; Pate, R. R.; Saunders, R.; Ward, D. S.; Dowda, M.; Felton, G. A Prospective Study of the Determinants of Physical Activity in Rural Fifth-Grade Children. *Prev Med* **1997**, *26* (2), 257–263.

Valois, R. F.; Dowda, M.; Trost, S. Cigarette Smoking Experimentation Among Rural Fifth Grade Students. *American Journal of Health Behavior* **1998**, *22* (2), 101–107.

Ward, D. S.; Trost, S. G.; Felton, G.; Saunders, R.; Parsons, M. A.; Dowda, M.; Pate, R. R. Physical Activity and Physical Fitness in African American Girls with and without Obesity. *Obesity Research* **1997**, 5(6), 572-577.

Weston, A. T.; Petosa, R.; Pate, R. R. Validation of an Instrument for Measurement of Physical Activity in Youth. *Med Sci Sports Exerc* **1997**, *29* (1), 138–143.

LEAP Articles

Colabianchi, N.; Dowda, M.; Pfeiffer, K. A.; Porter, D. E.; Almeida, M. J.; Pate, R. R. Towards an Understanding of Salient Neighborhood Boundaries: Adolescent Reports of an Easy Walking Distance and Convenient Driving Distance. *International Journal of Behavioral Nutrition and Physical Activity* **2007**, *4(1), 66*.

Dishman, R. K.; Hales, D. P.; Almeida, M. J.; Pfeiffer, K. A.; Dowda, M.; Pate, R. R. Factorial Validity and Invariance of the Physical Self-Description Questionnaire among Black and White Adolescent Girls. *Ethn Dis* **2006**, *16* (2), 551–558.

Dishman, R. K.; Hales, D. P.; Pfeiffer, K. A.; Felton, G. A.; Saunders, R.; Ward, D. S.; Dowda, M.; Pate, R. R. Physical Self-Concept and Self-Esteem Mediate Cross-Sectional Relations of Physical Activity and Sport Participation with Depression Symptoms among Adolescent Girls. *Health Psychology* **2006**, *25* (3), 396–407.

Dishman, R. K.; Motl, R. W.; Saunders, R.; Felton, G.; Ward, D. S.; Dowda, M.; Pate, R. R. Enjoyment mediates effects of school-based physical-activity intervention. *Medicine & Science in Sports & Exercise* **2005**, *37*(3), 478–487.

Dishman, R. K.; Motl, R. W.; Saunders, R. P.; Dowda, M.; Felton, G.; Ward, D. S.; Pate, R. R. Factorial Invariance and Latent Mean Structure of Questionnaires Measuring Social-Cognitive Determinants of Physical Activity among Black and White Adolescent Girls. *Preventive Medicine* **2001**, *34*, 100–108.

Dishman, R. K.; Saunders, R. P.; Felton, G.; Ward, D. S.; Dowda, M.; Pate, R. R. Goals and Intentions Mediate Efficacy Beliefs and Declining Physical Activity in High School Girls. *American Journal of Preventive Medicine* **2006**, *31* (6), 475–483.

Dishman, R. K.; Saunders, R. P.; Motl, R. W.; Dowda, M.; Pate, R. R. Self-Efficacy Moderated the Relation between Declines in Physical Activity and Perceived Social Support in High School Girls. *Journal of Pediatric Psychology* **2009**, *34(4), 441-451.*

Dowda, M.; Dishman, R. K.; Pfeiffer, K. A.; Pate, R. R. Family Support for Physical Activity in Girls from 8th to 12th Grade in South Carolina. *Preventive Medicine* **2007**, *44*, 153–159.

Dowda, M.; Dishman, R. K.; Porter, D.; Saunders, R. P.; Pate, R. R. Commercial Facilities, Social Cognitive Variables, and Physical Activity of 12th Grade Girls. *Ann Behav Med* **2009**, *37*(1), 77–87.

Dowda, M.; Pate, R. R.; Felton, G. M.; Saunders, R.; Ward, D. S.; Dishman, R. K.; Trost, S. G. Physical Activities and Sedentary Pursuits in African American and Caucasian Girls. *Research Quarterly for Exercise and Sport* **2004**, *75*(4), 352–360.

Dowda, M.; Pfeiffer, K. A.; Dishman, R.; Pate, R. R. Associations among Physical Activity, Health Indicators, and Employment in 12th Grade Girls. *Journal of Women's Health* **2007**, *16* (9), 1331–1339.

Dowda, M.; Pfeiffer, K. A.; Lobelo, F.; Porter, D. E.; Pate, R. R. Cardiorespiratory Fitness and Proximity to Commercial Physical Activity Facilities among 12th Grade Girls. *Journal of Adolescent Health* **2012**, *50(5), 497-502.*

Felton, G.; Dowda, M.; Ward, D. S.; Dishman, R. K.; Trost, S. G.; Saunders, R.; Pate, R. R. Differences in Physical Activity between Black and White Girls Living in Rural and Urban Areas. *Journal of School Health* **2002**, *72*(6), 250–255.

Felton, G.; Saunders, R. P.; Ward, D. S.; Dishman, R. K.; Dowda, M.; Pate, R. R. Promoting Physical Activity in Girls: A Case Study of One School's Success. *Journal of School Health* **2005**, *75(2), 57-62.*

Hales, D. P.; Dishman, R. K.; Motl, R. W.; Addy, C.; Pfeiffer, K. A.; Pate, R. R. Factorial Validity and Invariance of the Center for Epidemiologic Studies Depression (CES-D) Scale in a Sample of Black and White Adolescent Girls. *Ethnicity & Disease* **2006**, *16*, 1–8.

Horn, D. B.; O'Neill, J.R.; Pfeiffer, K.A.: Dowda, M.; Pate, R.R. Predictors of Physical Activity in the Transition after High School among Young Women. *J Phys Act Health* **2008**, *5*(2), 275–285.

Lobelo, F.; Dowda, M.; Pfeiffer, K. A.; Pate, R. R. Electronic Media Exposure and Its Association with Activity-Related Outcomes in Female Adolescents: Cross- Sectional and Longitudinal Analyses. *J Phys Act Health* **2009**, *6* (2), 137–143.

Motl, R. W.; Dishman, R. K.; Dowda, M.; Pate, R. R. Factorial Validity and Invariance of a Self-Report Measure of Physical Activity among Adolescent Girls. *Research Quarterly for Exercise and Sport* **2004**, *75*(3), 259–271.

Motl, R. W.; Dishman, R. K.; Felton, G.; Pate, R. R. Self-Motivation and Physical Activity among Black and White Adolescent Girls. *Medicine and Science in Sports and Exercise* **2003**,

35(1), 128–136.

Motl, R. W.; Dishman, R. K.; Saunders, R. P.; Dowda, M.; Pate, R. R. Perceptions of Physical and Social Environment Variables and Self-Efficacy as Correlates of Self-Reported Physical Activity among Adolescent Girls. *Journal of Pediatric Psychology* **2002**, *32*(1), 6–12.

Motl, R. W.; Dishman, R. K.; Saunders, R.; Dowda, M.; Felton, G.; Pate, R. R. Measuring Enjoyment of Physical Activity in Adolescent Girls. *American Journal of Preventive Medicine* **2001**, *21*(2), 110–117.

Motl, R. W.; Dishman, R. K.; Saunders, R. P.; Dowda, M.; Pate, R. R. Measuring Social Provisions for Physical Activity among Adolescent Black and White Girls. *Educational and Psychological Measurement* **2004**, *64*(4), 682–706.

Motl, R. W.; Dishman, R. K.; Trost, S. G.; Saunders, R. P.; Dowda, M.; Felton, G.; Ward, D. S.; Pate, R. R. Factorial validity and invariance of questionnaires measuring social-cognitive determinants of physical activity among adolescent girls. *Preventive Medicine* **2000**, *31*, 584–594.

Motl, R. W.; Dishman, R. K.; Ward, D. S.; Saunders, R. P.; Dowda, M.; Felton, G.; Pate, R. R. Examining Social-Cognitive Determinants of Intention and Physical Activity among Black and White Adolescent Girls Using Structural Equation Modeling. *Health Psychology* **2002**, *21*(5), 459–46.

Motl, R. W.; Dishman, R. K.; Ward, D. S.; Saunders, R. P.; Dowda, M.; Felton, G.; Pate, R. R. Comparison of Barriers Self-Efficacy and Perceived Behavioral Control for Explaining Physical Activity across 1 Year among Adolescent Girls. *Health Psychology* **2005**, *24*(1), 106-111.

Motl, R. W.; Dishman, R. K.; Ward, D. S.; Saunders, R. P.; Dowda, M.; Felton, G.; Pate, R. R. Perceived Physical Environment and Physical Activity across One Year among Adolescent Girls: Self Efficacy as a Possible Mediator? *Journal of Adolescent Health* **2005**, *37*(5), 403–408.

Pate, R. R.; Colabianchi, N.; Porter, D.; Almeida, M. J.; Lobelo, F.; Dowda, M. Physical Activity and Neighborhood Resources in High School Girls. *American Journal of Preventive Medicine* **2008**, *34(5), 413-419.*

Pate, R. R.; Dowda, M.; O'Neill, J.R; Ward, D.S. Change in Physical Activity Participation among Adolescent Girls from 8th to 12th Grade. *Journal of Physical Activity and Health* **2007**, *4*(1), 3–16.

Pate, R. R.; Ross, R.; Dowda, M.; Trost, S. G.; Sirard, J. Validation of a 3-Day Physical

Activity Recall Instrument in Female Youth. *Pediatric Exercise Science* **2003**, *15*, 257–265.

Pate, R. R.; Saunders, R.; Dishman, R. K.; Addy, C.; Dowda, M.; Ward, D. S. Long-Term Effects of a Physical Activity Intervention in High School Girls. *American Journal of Preventive Medicine* **2007**, *33(4), 276-280.*

Pate, R. R.; Ward, D. S.; O'Neill, J.R.; Dowda, M. Enrollment in Physical Education Is Associated with Overall Physical Activity in Adolescent Girls. *Research Quarterly for Exercise and Sport* **2007**, *78(4), 265-270.*

Pate, R. R.; Ward, D. S.; Saunders, R. P.; Felton, G.; Dishman, R. K.; Dowda, M. Promotion of Physical Activity in High School Girls: A Randomized Controlled Trial. *American Journal Public Health* **2005**, *95*(9), 1582–1587.

Pfeiffer, K. A.; Colabianchi, N.; Dowda, M.; Porter, D.; Hibbert, J.; Pate, R. R. Examining the Role of Churches in Adolescent Girls' Physical Activity. *J Phys Act Health* **2011**, *8*, 227–233.

Pfeiffer, K. A.; Dowda, M.; Dishman, R. K.; McIver, K. L.; Sirard, J.R.; Ward, D.S.; Pate, R.R. Sport Participation and Physical Activity in Adolescent Females across a Four-Year Period. *Journal of Adolescent Health* **2006**, *39*, 523–529.

Saunders, R. P.; Motl, R. W.; M., D.; Dishman, R. K.; Pate, R. R. Comparison of Social Variables for Understanding Physical Activity in Adolescent Girls. *American Journal of Health Behavior* **2004**, *28* (5), 426–436.

Saunders, R. P.; Pate, R. R.; Dowda, M.; Ward, D. S.; Epping, J. N.; Dishman, R. K. Assessing Sustainability of Lifestyle Education for Activity Program (LEAP. *Health Education Research* **2012**, *27* (2), 319–330.

Saunders, R. P.; Ward, D.; Felton, G. M.; Dowda, M.; Pate, R. R. Examining the Link between Program Implementation and Behavior Outcomes in the Lifestyle Education for Activity Program (LEAP. *Evaluation and Program Planning* **2006**, *29*, 352–364.

Sirard, J.R.; Pfeiffer, K.A.; Dowda, M.; Pate, R.R. Race Differences in Activity, Fitness, and BMI in Female Eighth Graders Categorized by Sports Participation Status. *Pediatric Exercise Science* **2008**, *20* (2), 198–210.

Trilk, J. L.; Ward, D. S.; Dowda, M.; Pfeiffer, K. A.; Porter, D. E.; Hibbert, J.; Pate, R. R. Do Physical Activity Facilities near Schools Affect Physical Activity in High School Girls? *Health & Place* **2011**, *17(2), 651-657.*

Trost, S. G.; Pate, R. R.; Dowda, M.; Ward, D. S.; Felton, G.; Saunders, R. Psychosocial

Correlates of Physical Activity in White and African-American Girls. *Journal of Adolescent Health* **2002**, *31*(3), 226–233.

Ward, D. S.; Dowda, M.; Trost, S. G.; Felton, G. M.; Dishman, R. K.; Pate, R. R. Physical Activity Correlates in Adolescent Girls Who Differ by Weight Status. *Obesity* **2006**, *14*(1), 97–105.

Ward, D. S.; Saunders, R.; Felton, G. M.; Williams, E.; Epping, J. N.; Pate, R. R. Implementation of a School Environment Intervention to Increase Physical Activity in High School Girls. *Health Education Research* **2006**, *21*(6), 896–910.

TAAG Articles

Cohen, D.; Scott, M.; Wang, F. Z.; McKenzie, T. L.; Porter, D. School Design and Physical Activity among Middle School Girls. *J Phys Act Health* **2008** *5,* 719-731.

Cohen, D. A.; Ashwood, J. S.; Scott, M. M.; Overton, A.; Evenson, K. R.; Staten, L. K.; Porter, D.; McKenzie, T. L.; Catellier, D. Public parks and physical activity among adolescent girls. *Pediatrics* **2006**, 118(5), e1381-9.

Dishman, R. K.; Motl, R. W.; Sallis, J. F.; Dunn, A. L.; Birnbaum, A. S.; Welk, G. J.; Bedimo-Rung, A. L.; Voorhees, C. V.; Jobe, J. B. Self-Management Strategies Mediate Self-Efficacy and Physical Activity. *Am J Prev Med* **2005**, *29*, 10–18.

Dowda, M.; McKenzie, T. L.; Cohen, D. A.; Scott, M. M.; Evenson, K. R.; Bedimo-Rung, A. L.; Voorhees, C. C.; Almeida, M. J. Commercial Venues as Supports for Physical Activity in Adolescent Girls.*Prev Med*, **2007**, *45(2-3), 163-168.*

Elder, J. P.; Lytle, L.; Sallis, J. F.; Young, D. R.; Steckler, A.; Simons-Morton, D.; Stone, E.; Jobe, J. B.; Stevens, J.; Lohman, T.; Webber, L.; Pate, R.; Saksvig, B. I.; Ribisl, K. A description of the social-ecological framework used in the trial of activity for adolescent girls (TAAG).*Health Educ Res*, **2007**, *22(2), 155-65.*

Kelly, E. B.; Parra-Medina, D.; Pfeiffer, K. A.; Dowda, M.; Conway, T.; Webber, L.; Jobe, J.; Going, S.; Pate, R. R. Correlates of Physical Activity in Black, Hispanic, and White Middle School Girls. *J Phys Act Health* **2010**, *7*(2), 184–193.

Lohman, T. G.; Ring, K.; Pfeiffer, K.; Camhi, S.; Arredondo, E.; Pratt, C.; Pate, R. R.; Webber, L. S. Relationships among Fitness, Body Composition, and Physical Activity. *Med Sci Sports Exerc* **2008**, *40* (6), 1163–1170.

Pate, R. R.; Sallis, J. F.; Ward, D. S.; Stevens, J.; Dowda, M.; Welk, G. J.; Young, D. R.; Jobe, J. B.; Strikmiller, P. K. Age-Related Changes in Types and Contexts of Physical

Activity in Middle School Girls. *Am J Prev Med* **2010**, *39*(5), 433–439.

Pate, R. R.; Stevens, J.; Pratt, C.; Sallis, J. F.; Schmitz, K. H.; Webber, L. S.; Welk, G. Young DR. Objectively Measured Physical Activity in Sixth-Grade Girls. *Arch Pediatr Adoles Med*, 2006; *160*, 1262-1268.

Pate, R. R.; Stevens, J.; Webber, L. S.; Dowda, M.; Murray, D. M.; Young, D. R.; Going, S. Age-related change in physical activity in adolescent girls. *Journal of Adolescent Health* **2009**, *44*, 275–282.

Pfeiffer, K. A.; Schmitz, K. H.; McMurray, R. G.; Treuth, M. S.; Murray, D. A.; Pate, R. R. Physical Activities in Adolescent Girls: Variability in Energy Expenditure. *American Journal of Preventive Medicine* **2006**, *31* (4).

Saksvig, B. I.; Catellier, D. J.; Pfeiffer, K.; Schmitz, K. H.; Conway, T.; Going, S.; Ward, D.; Strikmiller, P.; Treuth, M. S. *Travel by Walking before and after School and Physical Activity among Adolescent Girls*; Arch Pediatr Adolesc Med, 2007; 161(2), 153-158.

Saunders, R. P.; Moody, J. Community Agency Survey Formative Research Results from the TAAG Study. *Health Educ Behav* **2006**, *33*(1), 12–24.

Taverno Ross, S.E.; Dowda, M.; Beets, M.W.; Pate, R.R. Physical Activity Behavior and Related Characteristics of Highly Active Eight-Grade Girls. *Journal of Adolescent Health* **2013**, *52*, 745–751.

Stevens, J.; Murray, D. M.; Baggett, C. D.; Elder, J. P.; Lohman, T. G.; Lytle, L. A.; Pate, R. R.; Pratt, C. A.; Treuth, M. S.; Webber, L. S.; Young, D. R. Objectively Assessed Associations between Physical Activity and Body Composition in Middle-School Girls: The Trial of Activity for Adolescent Girls. *Am J Epidemiol* **2007**, *166(11), 1298-305.*

Treuth, M. S.; Catellier, D. J.; Schmitz, K.; Pate, R. R.; Elder, J. P.; McMurray, R. G.; Blew, R. M.; Yang, S.; Webber, L. Weekend and Weekday Patterns of Physical Activity in Overweight and Normal-Weight Adolescent Girls. *Obesity* **2007**, *15*, 1782–1788.

Treuth, M. S.; Schmitz, K.; Catellier, D. J.; McMurray, R. G.; Murray, D. M.; Aleida, M. J.; Going, S.; Norman, J. E.; Pate, R. Defining Accelerometer Thresholds for Activity Intensities in Adolescent Girls. *MSSE* **2004**, *36*, 1259–1266.

Trilk, J. L.; Pate, R. R.; Pfeiffer, K. A.; Dowda, M.; Addy, C. L.; Ribisl, K. M.; Neumark-Sztainer, D.; Lytle, L. A. A Cluster Analysis of Physcal Activity and Sedentary Behavior Patterns in Middle School Girls. *Journal of Adolescent Health* **2012**, *51*, 292–298.

Vu, M. B.; Murrie, D.; Gonzalez, V.; Jobe, J. B. Listening to Girls and Boys Talk about Girls'

Physical Activity Behaviors. *Health Educ Behav* **2006,** *33(1):81-96.*

Webber, L. S.; Catellier, D. J.; Lytle, L. A.; Murray, D. M.; Pratt, C. A.; Young, D. R.; Elder, J. P.; Lohman, T. G.; Stevens, J.; Jobe, J. B.; Pate, R. R. Promoting Physical Activity in Middle School Girls: Trial of Activity for Adolescent Girls. *American Journal of Preventive Medicine* **2008**, *34* (3), 173–184.

Young, D. R.; Felton, G. M.; Grieser, M.; Elder, J. P.; Johnson, C.; Lee, J. S.; Kubik, M. Y. Policies and Opportunities for Physical Activity in Middle School Environments. *J Sch Health* **2007**, *77*, 41-47.

CHAMPS Articles

Addy, C. L.; Trilk, J. L.; Dowda, M.; Byun, W.; Pate, R. R. Assessing Preschool Children's Physical Activity: How Many Days of Accelerometry Measurement. *Pediatric Exercise Science* **2014**, *26(1):103-109.*

Beets, M. W.; Bornstein, D.; Dowda, M.; Pate, R. R. Compliance with National Guidelines for Physical Activity in US Preschoolers: Measurement and Interpretation. *Pediatrics* **2011**, *127*, 658-64.

Bornstein, D. B.; Beets, M. W.; Byun, W.; Welk, G.; Bottai, M.; Dowda, M.; Pate, R. Equating Accelerometer Estimates of Moderate-to-Vigorous Physical Activity: In Search of the Rosetta Stone. *Journal of Science and Medicine in Sport* **2011**, *14(5), 404-410.*

Brown, W. H.; Pfeiffer, K. A.; McIver, K. L.; Dowda, M.; Almeida, J. M.; Pate, R. R. Assessing Preschool Children's Physical Activity: The Observational System for Recording Physical Activity in Children-Preschool Version. *Research Quarterly for Exercise and sport* **2006**, *77(2), 167-176.*

Brown, W. H.; Pfeiffer, K. A.; McIver, K. L.; Dowda, M.; Addy, C. L.; Pate, R. R. Social and Environmental Factors Associated with Preschoolers' Nonsedentary Physical Activity. *Child Development* **2009**, *80*, 45-58.

Byun, W.; Beets, M. W.; Pate, R. R. Sedentary Behavior in Preschoolers: How Many Days of Accelerometer Monitoring Is Needed? *International Journal of Environmental Research and Public Health* **2015**, *12(10), 13148-13161.*

Byun, W.; Dowda, M.; Pate, R. R. Correlates of Objectively Measured Sedentary Behavior in US Preschool Children. *Pediatrics* **2011**, 128(5), 937-945.

Byun, W.; Liu, J.; Pate, R. R. Association between Objectively Measured Sedentary Behavior and Body Mass Index in Preschool Children. *International Journal of Obesity* **2013**, *37*, 961-

965.

Dowda, M.; Brown, W. H.; McIver, K. L.; Pfeiffer, K. A.; O'Neill, J.R.; Addy, C.L.; Pate, R.R. Policies and Characteristics of the Preschool Environment and Physical Activity of Young Children. *Pediatrics* **2009**, *123(2):e261-6.*

Dowda, M.; Pfeiffer, K. A.; Brown, W. H.; Mitchell, J. A.; Byun, W.; Pate, R. R. Parental and environmental correlates of physical activity of children attending preschool. *Archives of Pediatrics & Adolescent Medicine* **2011**,*165(10), 939-44.*

Howie, E. K.; Brown, W. H.; Dowda, M.; McIver, K. L.; Pate, R. R. Physical Activity Behaviours of Highly Active Preschoolers. *Pediatric Obesity* **2013**, *8(2),142-9.*

McIver, K. L.; Brown, W. H.; Pfeiffer, K. A.; Dowda, M.; Pate, R. R. Assessing Children's Physical Activity in Their Homes: The Observational System for Recording Physical Activity in Children-home. *Journal of Applied Behavior Analysis* **2009**, *42*, 1-6.

McIver, K. L.; Brown, W. H.; Pfeiffer, K. A.; Dowda, M.; Pate, R. R. Development and Testing of the Observational System for Recording Physical Activity in Children: Elementary School. *Research Quarterly for Exercise and Sport* **2016**, *87(1), 101-9.*

O'Neill, J.R.; Williams, H.G.; Pfeiffer, K.A.; Dowda, M.; McIver, K.L.; Brown, W.H.; Pate, R.R.. Young Children's Motor Skill Performance: Relationships with Activity Types and Parent Perception of Athletic Competence. *Journal of Science and Medicine in Sport* **2014**, *17(6), 607-610.*

Pate, R. R.; Almeida, M. J.; McIver, K. L.; Pfeiffer, K. A.; Dowda, M. Validation and Calibration of an Accelerometer in Preschool Children. *Obesity* **2006**, 14(11), 2000-2006.

Pate, R. R.; Dowda, M.; Brown, W. H.; Mitchell, J.; Addy, C. Physical Activity in Preschool Children with the Transition to Outdoors. *Journal of Physical Activity and Health* **2013**, *10(2), 170-175.*

Pate, R. R.; McIver, K.; Dowda, M.; Brown, W. H.; Addy, C. Directly Observed Physical Activity Levels in Preschool Children. *Journal of School Health* **2008**, *78(8), 438-444.*

Pate, R. R.; O'Neill, B., J.R.; Brown, W.H.; Pfeiffer, K.A.; Dowda, M.; Addy, C.. Prevalence of Compliance with a New Physical Activity Guideline for Preschool-Age Children. *Childhood Obesity* **2015**, *11(4), 415-420.*

Pfeiffer, K. A.; Dowda, M.; McIver, K. L.; Pate, R. R. Factors Related to Objectively Measured Physical Activity in Preschool Children. *Pediatric Exercise Science* **2009**, *21,* 196-208.

Pfeiffer, K. A.; McIver, K. L.; Dowda, M.; Almeida, M. J.; Pate, R. R. Validation and Calibration of the Actical Accelerometer in Preschool Children. *Medicine and Science in Sports and Exercise* **2006,** *38*, 152-157.

Schenkelberg, M. A.; O'Neill, J.R.; Dowda, M.; Pate, R.R. How Does the Relationship Between Motor Skill Performance and Body Mass Index Impact Physical Activity in Preschool Children? *Pediatric Exercise Science* **2018**, *30(2), 266-72.*

True, L.; Pfeiffer, K. A.; Dowda, M.; Williams, H. G.; Brown, W. H.; O'Neill, J.R.; Pate, R.R. Motor Competence and Characteristics within the Preschool Environment. *Journal of Science and Medicine in Sports* **2017**, 20 751-755.

Williams, H. G.; Pfeiffer, K. A.; Dowda, M.; Jeter, C.; Jones, S.; Pate, R. R. A Field-Based Testing Protocol for Assessing Gross Motor Skills in Preschool Children: The Children's Activity and Movement in Preschool Study Motor Skills Protocol. *Measurement in Physical Education and Exercise Science* **2009**, *13(3), 151-165.*

Williams, H. G.; Pfeiffer, K. A.; O'Neill, J.R.; Dowda, M.; McIver, K.L; Brown, W.H.; Pate, R.R. Motor Skill Performance and Physical Activity in Preschool Children. *Obesity* **2008**, *16*, 1421-1426.

SHAPES Articles

Byun, W.; Blair, S. N.; Pate, R. R. Objectively Measured Sedentary Behavior in Preschool Children: Comparison between Montessori and Traditional Preschools. *International Journal of Behavioral Nutrition and Physical Activity* **2013**, *Dec;10(1), 1-7.*

España-Romero, V.; Mitchell, J. A.; Dowda, M.; O'Neill, JR; Pate, R.R. Objectively Measured Sedentary Time, Physical Activity and Markers of Body Fat in Preschool Children. *Pediatric Exercise Science* **2013**, *25(1), 154-163.*

Howie, E. K.; Brewer, A.; Brown, W. H.; Pfeiffer, K. A.; Saunders, R. P.; Pate, R. R. The 3-Year Evolution of a Preschool Physical Activity Intervention through a Collaborative Partnership between Research Interventionists and Preschool Teachers. *Health Education Research* **2014**, *29(3), 491-502.*

Howie, E. K.; Brewer, A. E.; Brown, W. H.; Saunders, R. P.; Pate, R. R. Systematic Dissemination of a Preschool Physical Activity Intervention to the Control Preschools. *Evaluation and Program Planning* **2016**, *57*, 1-7.

Howie, E. K.; Brewer, A. E.; Dowda, M.; McIver, K. L.; Saunders, R. P.; Pate, R. R. A Tale

of 2 Teachers: A Preschool Physical Activity Intervention Case Study. *Journal of school health* **2016**, 86, 23-30.

Kennedy, A. B.; Schenkelberg, M.; Moyer, C.; Pate, R.; Saunders, R. P. Process Evaluation of a Preschool Physical Activity Intervention Using Web-Based Delivery. *Evaluation and Program Planning* **2017**, *60*, 24–36.

Lau, E. Y.; Saunders, R. P.; Beets, M. W.; Cai, B.; Pate, R. R. Factors Influencing Implementation of a Preschool-Based Physical Activity Intervention. *Health education research* **2017**, *32(1), 69-80.*

O'Neill, J.R.; Pfeiffer, K.A.; Dowda, M.; Pate. R.R. In-School and out-of-School Physical Activity in Preschool Children. *Journal of Physical Activity and Health* **2016**, *6*, 606-610.

Pate, R. R.; Brown, W. H.; Pfeiffer, K. A.; Howie, E. K.; Saunders, R. P.; Addy, C. L.; Dowda, M. An Intervention to Increase Physical Activity in Children: A Randomized Controlled Trial with 4-Year-Olds in Preschools. *American journal of preventive medicine* **2016**, *51(1), 12-22.*

Pate, R. R.; O'Neill, J.R.; Byun, W.; McIver, K.L.; Dowda, M.; Brown, W.H. .Physical Activity in Preschool Children: Comparison between Montessori and Traditional Preschools. *Journal of School Health* **2014**, *84(11), 716-721.*

Pfeiffer, K. A.; Saunders, R. P.; Brown, W. H.; Dowda, M.; Addy, C. L.; Pate, R. R. Study of Health and Activity in Preschool Environments (SHAPES): Study Protocol for a Randomized Trial Evaluating a Multi-Component Physical Activity Intervention in Preschool Children. *BMC Public Health*, 2013; 13(1), 1-8.

Ross, S. T.; Dowda, M.; Saunders, R.; Pate, R. Double Dose: The Cumulative Effect of TV Viewing at Home and in Preschool on Children's Activity Patterns and Weight Status. *Pediatric Exercise Science* **2013**, 25, 262-272.

Saunders, R. P.; Dowda, M.; Pfeiffer, K. A.; Brown, W. H.; Pate, R. R. Childcare Center Characteristics Moderate the Effects of a Physical Activity Intervention. *International Journal of Environmental Research and Public Health* **2020**, *17* (1), 101.

Saunders, R. P.; Pfeiffer, K.; Brown, W. H.; Howie, E. K.; Dowda, M.; O'Neill, J.R.; McIver, K.; Pate, R.R., Pate RR. Evaluating and Refining the Conceptual Model Used in the Study of Health and Activity in Preschool Environments (SHAPES) Intervention. *Health Education & Behavior* **2017**, *44(6), 876-84.*

Saunders, R. P.; Schenkelberg, M. A.; Moyer, C.; Howie, E. K.; Brown, W. H.; Pate, R. R. The Translation of an Evidence-Based Preschool Physical Activity Intervention from in-

Person to Online Delivery of Professional Development to Preschool Teachers. *Translational Behavioral Medicine* **2019**, *9*(6), 1186–1196.

TRACK Articles

Barr-Anderson, D. J.; Flynn, J. I.; Dowda, M.; Ross, S. E.; Schenkelberg, M. A.; Reid, L. A.; Pate, R. R. The Modifying Effects of Race/Ethnicity and Socioeconomic Status on the Change in Physical Activity from Elementary to Middle School. *Journal of Adolescent Health* **2017**, *61(5), 562-70.*

Bucko, A. G.; Porter, D. E.; Saunders, R.; Shirley, L.; Dowda, M.; Pate, R. R. Walkability Indices and Children's Walking Behavior in Rural vs. Urban Areas. *Health & Place* **2021**, *72*, 102707.

Clennin, M.; Brown, A.; Lian, M.; Dowda, M.; Colabianchi, N.; Pate, R. R. Neighborhood Socioeconomic Deprivation Associated with Fat Mass and Weight Status in Youth. *International Journal of Environmental Research and Public Health* **2020**, *17* (17), 6421.

Clennin, M. N.; Lian, M.; Colabianchi, N.; Kaczynski, A.; Dowda, M.; Pate, R. R. Associations among Neighborhood Socioeconomic Deprivation, Physical Activity Facilities, and Physical Activity in Youth during the Transition from Childhood to Adolescence. *International Journal of Environmental Research and Public Health* **2019**, *16*(19), 3703.

Colabianchi, N.; Clennin, M. N.; Dowda, M.; McIver, K. L.; Dishman, R. K.; Porter, D. E.; Pate, R. R. Moderating Effect of the Neighborhood Physical Activity Environment on the Relation between Psychosocial Factors and Physical Activity in Children: A Longitudinal Study. *J Epidemiol Community Health* **2019**, *73*, 598–604.

Colabianchi, N.; Griffin, J. L.; McIver, K. L.; Dowda, M.; Pate, R. R. Where Are Children Active and Does It Matter for Physical Activity? A Latent Transition Analysis. *Journal of Physical Activity and Health* **2016**, *13(12), 294-300.*

Dishman, R. K., Saunders, R. P., McIver, K. L., Dowda, M., & Pate, R. R. (2013). Construct validity of selected measures of physical activity beliefs and motives in fifth and sixth grade boys and girls. *Journal of Pediatric Psychology* **2013***, 38*(5), 563-576.

Dishman, R. K.; Dowda, M.; McIver, K. L.; Saunders, R. P.; Pate, R. R. Naturally-Occurring Changes in Social-Cognitive Factors Modify Change in Physical Activity during Early Adolescence. *PloS One* **2017**, *10;12(2), e0172040.*

Dishman, R. K.; McIver, K. L.; Dowda, M.; Saunders, R. P.; Pate, R. R. Motivation and Behavioral Regulation of Physical Activity in Middle-School Students. *Medicine and Science in Sports and Exercise* **2015**, 47, 1913.

Dishman, R. K.; McIver, K. L.; Dowda, M.; Pate, R. R. Declining Physical Activity and Motivation from Middle School to High School. *Medicine and Science in Sports and Exercise* **2018**, 50, 1206-1215.

Dishman, R. K.; McIver, K. L.; Dowda, M.; Saunders, R. P.; Pate, R. R. Self-Efficacy, Beliefs, and Goals: Moderation of Declining Physical Activity during Adolescence. *Health Psychol* **2019**, *38*, 483–493.

Dowda, M.; Dishman, R. K.; Saunders, R. P.; Pate, R. R. Associations between Three Measures of Physical Activity and Selected Influences on Physical Activity in Youth Transitioning from Elementary to Middle School. *Sports Medicine and Health Science* **2021**, *3*, 21–27.

Dowda, M.; Saunders, R. P.; Colabianchi, N.; Dishman, R. K.; McIver, K. L.; Pate, R. R. Longitudinal Associations between Psychosocial, Home, and Neighborhood Factors and Children's Physical Activity. *Journal of Physical Activity and Health* **2020**, *17*, 306–312.

Dowda, M.; Taverno Ross, S. E.; McIver, K. L.; Dishman, R. K.; Pate, R. R. Physical Activity and Changes in Adiposity in the Transition from Elementary to Middle School. *Childhood Obesity* **2017**, 1*3(1), 53-62.*

Forthofer, M.; Dowda, M.; McIver, K.; Barr-Anderson, D. J.; Pate, R. Associations between Maternal Support and Physical Activity among 5th Grade Students. *Maternal and child health journal* **2016**, *20(3), 720-729.*

Forthofer, M.; Dowda, M.; O'Neill, J.R.; Addy, C.L.; McDonald, S.; Reid, L.; Pate, R.R. Effect of Child Gender and Psychosocial Factors on Physical Activity From Fifth to Sixth Grade. *Journal of Physical Activity and Health* **2017**, *14(12), 953-958.*

Lau, E. Y.; Barr-Anderson, D. J.; Dowda, M.; Forthofer, M.; Saunders, R. P.; Pate, R. R. Associations between Home Environment and After-School Physical Activity and Sedentary Time among 6th Grade Children. *Pediatric Exercise Science* **2015**, 27, 226-233.

Lau, E. Y.; Dowda, M.; McIver, K. L.; Pate, R. R. Changes in Physical Activity in the School, Afterschool, and Evening Periods during the Transition from Elementary to Middle School. *Journal of School Health* **2017**, 87, 531-537.

McDonald, S.; Dowda, M.; Colabianchi, N.; Porter, D.; Dishman, R. K.; Pate, R. R. Perceptions of the Neighborhood Environment and Children's Afterschool Moderate-to-Vigorous Physical Activity. *Pediatric Exercise Science* **2015**, 27, 243-251.

Pate, R.R.; Dowda, M.: Dishman, R.K.; Colabianchi, N.; Saunders, R.P.; McIver, K.L.

Change in Children's Physical Activity: Predictors in the Transition from Elementary to Middle School. *Am J Prev Med* **2019**, *56:e65-e73.*

Pate, R. R.; Dowda, M.; Dishman, R. K.; Saunders, R. P.; Cordan, K. L.; Shull, E. R.; Bucko, A. G.; Colabianchi, N. Determinants of Change in Physical Activity in Children during the Transition from Elementary to High School. *Under Review* **2022**.

Pate, R. R.; Dowda, M.; Dishman, R. K.; Gorab, J.; Saunders, R. P. Longitudinal Association of Biological Maturation with Physical Activity Behaviors in Girls Transitioning from 5th to 7th Grade. *Am J Hum Biol* **2022**, e23737.

Pate, R. R.; Dowda, M.; Saunders, R. P.; Colabianchi, N.; Clennin, M. N.; Cordan, K. L.; Shirley, W. L. Operationalizing and Testing the Concept of a Physical Activity Desert. *Journal of Physical Activity and Health* **2021**, *18*(5), 533–540.

Pate, R. R.; Saunders, R. P.; Taverno Ross, S. E.; Dowda, M. Patterns of Age- Related Change in Physical Activity during the Transition from Elementary to High School. *Preventive Medicine Reports* **2022**, *26*, 101712.

Pate, R. R.; Schenkelberg, M. A.; Dowda, M.; McIver, K. L. Group Based Physical Activity Trajectories in Children Transitioning from Elementary to High School. *BMC Public Health* **2019**, *19*(1), 1-7.

Ross, S. E.; Clennin, M. N.; Dowda, M.; Colabianchi, N.; Pate, R. R. Stepping It Up: Walking Behaviors in Children Transitioning from 5th to 7th Grade. *International Journal of Environmental Research and Public Health* **2018**, *15(2), 262.*

Ross, S. E. T.; Byun, W.; Dowda, M.; McIver, K. L.; Saunders, R. P.; Pate, R. R. Sedentary Behaviors in Fifth-Grade Boys and Girls: Where, with Whom, and Why? *Childhood Obesity* **2013**, *9*, 532–539.

Ross, S. E. T.; Militello, G.; Dowda, M.; Pate, R. R. Changes in Diet Quality in Youth Living in South Carolina From Fifth to 11th Grade. *Journal of Nutrition Education and Behavior* **2020**, *52*(10), 928–934.

Ross, T.; Sharon, E.; Dowda, M.; Dishman, R. K.; Pate, R. R. Classes of Physical Activity and Sedentary Behavior in 5th Grade Children. *American Journal of Health Behavior* **2016**, *40(3), 352-361.*

Saunders, R. P.; Dishman, R. K.; Dowda, M.; Pate, R. R. Personal, Social, and Environmental Influences on Physical Activity in Groups of Children as Defined by Different Physical Activity Patterns. *Journal of Physical Activity and Health* **2020**, *17*(9), 867-873.

Saunders, R. P.; Dowda, M.; McIver, K.; McDonald, S. M.; Pate, R. R. Physical and Social Contexts of Physical Activity Behaviors of Fifth and Seventh Grade Youth. *Journal of School Health* **2018**, *88(2), 122-31.*

Shull, E. R.; Dowda, M.; Saunders, R. P.; McIver, K.; Pate, R. R. Sport Participation, Physical Activity and Sedentary Behavior in the Transition from Middle School to High School. *Journal of Science and Medicine in Sport* **2020**, *23*, 385–389.

Taverno Ross, S. E.; Dowda, M.; Colabianchi, N.; Saunders, R.; Pate, R.R. After-School Setting, Physical Activity, and Sedentary Behavior in 5th Grade Boys and Girls. *Health and Place* **2012**, *18*, 951–955.
